# Identification of allosteric fingerprints of alpha-synuclein aggregates in matrix metalloprotease-1 and substrate-specific virtual screening with single molecule insights

**DOI:** 10.1038/s41598-022-09866-7

**Published:** 2022-04-06

**Authors:** Sumaer Kamboj, Chase Harms, Derek Wright, Anthony Nash, Lokender Kumar, Judith Klein-Seetharaman, Susanta K. Sarkar

**Affiliations:** 1 Department of Physics, Colorado School of Mines, Golden, CO USA; 2 Nuffield Department of Clinical Neurosciences, University of Oxford, Oxford, UK; 3 Department of Chemistry, Colorado School of Mines, Golden, CO USA

**Keywords:** Biophysics, Computational biology and bioinformatics

## Abstract

Alpha-synuclein (aSyn) has implications in pathological protein aggregations in neurodegeneration. Matrix metalloproteases (MMPs) are broad-spectrum proteases and cleave aSyn, leading to aggregation. Previous reports showed that allosteric communications between the two domains of MMP1 on collagen fibril and fibrin depend on substrates, activity, and ligands. This paper reports quantification of allostery using single molecule measurements of MMP1 dynamics on aSyn-induced aggregates by calculating Forster Resonance Energy Transfer (FRET) between two dyes attached to the catalytic and hemopexin domains of MMP1. The two domains of MMP1 prefer open conformations that are inhibited by a single point mutation E219Q of MMP1 and tetracycline, an MMP inhibitor. A two-state Poisson process describes the interdomain dynamics, where the two states and kinetic rates of interconversion between them are obtained from histograms and autocorrelations of FRET values. Since a crystal structure of aSyn-bound MMP1 is unavailable, binding poses were predicted by molecular docking of MMP1 with aSyn using ClusPro. MMP1 dynamics were simulated using predicted binding poses and compared with the experimental interdomain dynamics to identify an appropriate pose. The selected aSyn-MMP1 binding pose near aSyn residue K45 was simulated and analyzed to define conformational changes at the catalytic site. Allosteric residues in aSyn-bound MMP1 exhibiting strong correlations with the catalytic motif residues were compared with allosteric residues in free MMP1, and aSyn-specific residues were identified. The allosteric residues in aSyn-bound MMP1 are K281, T283, G292, G327, L328, E329, R337, F343, G345, N346, Y348, G353, Q354, D363, Y365, S366, S367, F368, P371, R372, V374, K375, A379, F391, A394, R399, M414, F419, V426, and C466. Shannon entropy was defined to quantify MMP1 dynamics. Virtual screening was performed against a site on selected aSyn-MMP1 binding poses, which showed that lead molecules differ between free MMP1 and substrate-bound MMP1. Also, identifying aSyn-specific allosteric residues in MMP1 enabled further selection of lead molecules. In other words, virtual screening needs to take substrates into account for potential substrate-specific control of MMP1 activity in the future. Molecular understanding of interactions between MMP1 and aSyn-induced aggregates may open up the possibility of degrading aggregates by targeting MMPs.

## Introduction

Lewy bodies, intraneuronal cytoplasmic protein aggregates, are commonly observed in the brains of patients with Alzheimer's disease (AD) and Parkinson's disease (PD)^1^. Alpha-synuclein (aSyn) is often the common protein detected in Lewy bodies observed in AD and PD^2^. An established body of evidence shows that the C-terminal truncation of aSyn by proteases leads to protein aggregation and Lewy body formation. Both intracellular and extracellular proteolytic systems take part in the breakup of aSyn^3^. The partial cleavage of aSyn explains aggregates due to fibrillation^4^ and misfolding^5,6^. Although aggregates could be a consequence instead of a cause of AD and PD^7,8^, the uncontrolled aggregation will lead to cell death and worsening of effects^9,10^. As such, there is a need to find ways to degrade existing aggregates.

A growing body of evidence shows that matrix metalloproteases (MMPs) can partially cleave aSyn^11–15^. Prior reports have shown that MMP1, MMP2, MMP3, MMP9, and MT1-MMP can partially cleave aSyn^13,14^ with the possible cleavage sites identified^13^. MMPs are broad-spectrum proteases known to degrade extracellular matrix and non-matrix proteins^16,17^, but a growing body of evidence suggests proteolytic and non-proteolytic intracellular functions of MMPs^18^. MMPs are found in extracellular space^19^, intracellular space^20^, blood^21^, intestine^22^, brain^23^ and can alter the blood–brain barrier^24^. The implication of MMPs in partial cleavage of aSyn is significant because tetracycline^25,26^, a well-known inhibitor of MMPs^27^, has shown therapeutic potential in AD^28^ and PD^29^. Thus, the broad-spectrum protease activity of MMPs leads to aSyn-induced aggregation. It might also be useful to leverage MMP activity to do the opposite of aggregation, i.e., degrade preexisting aSyn-induced aggregates. However, it is not clear how MMPs might affect aSyn-induced aggregates, which are water-insoluble and challenging to study using standard biochemical assays. To this end, we have developed a single molecule tracking approach and weight-based activity assay to study water-soluble MMPs interacting with water-insoluble substrates. As such, it is possible to study MMP activity on aSyn-induced aggregates at the single molecule level.

However, it is not enough to know how MMP interacts with aggregates. Due to broad-spectrum activity, targeting MMPs for improving human health is challenging because MMPs interact with and degrade many proteins in the human body^30^. Due to such diverse functions, any drug used for inhibiting MMPs results in adverse side effects^31^. As such, there is only one FDA-approved MMP inhibitor (doxycycline hyclate)^32^. To circumvent this problem, we need to understand the mechanisms behind diverse functions. The catalytic domain sequence of MMP1 is very similar to other MMPs in the family. However, the hemopexin domain sequence varies^33,34^, suggesting differences in activity and substrate specificity among MMPs due to long-distance communications^17,35^. Recently, we reported activity-dependent MMP1 dynamics and allosteric communications on type-1 collagen fibrils^36^ and fibrin^37^. We showed that functionally relevant conformations have the catalytic and hemopexin domains of MMP1 well-separated. These open conformations often accompany a larger catalytic pocket opening of MMP1, enabling the substrates to get closer to the active site of MMP1. Building on this, we have the opportunity to define dynamics and allosteric communications of MMP1 on aSyn-induced aggregates.

Quantifying dynamics and allosteric communications of MMP1 on a substrate involves quantifying randomness because proteins are inherently flexible biomolecules having both intra- and interdomain motions. Two types of randomness appear as proteins interact with their surroundings and substrates. First, the amino acids' locations and angles have a spatially random component at a particular time point. Second, as time progresses, proteins sample through different conformations leading to temporal randomness. Such correlated or collective fluctuations are essential for functions, including allosteric regulation^38–40^, generation of mechanical work^41,42^, catalysis^43^, ligand binding^44^, and protein folding^45^. Correlated motions decrease the conformational entropy and affect the kinetics and thermodynamics of biological processes^46^. Shannon entropy is a quantitative measure to describe randomness in computer science and pattern recognition and quantify allostery in proteins to gain insights into their functional relevance^47–50^.

In this paper, we report measurements of activity-dependent MMP1 interdomain dynamics on aSyn-induced aggregates of *E. coli* proteins at the single molecule level. MMP1 prefers open conformations on aSyn-induced aggregates, and these conformations are inhibited by tetracycline. A two-state Poisson process describes the interdomain dynamics of MMP1 on aggregates. Since aSyn-bound MMP1 crystal structures are not available, we used ClusPro for molecular docking of MMP1 with aSyn. We performed all-atom simulations of different binding poses to calculate the interdomain dynamics and compare them with experimental dynamics. It appears that the pose with MMP1 bound to the middle U-shaped region of aSyn structure leads to dynamics similar to experimental dynamics. We used this pose to quantify allostery, define changes at the catalytic site, and identify functionally relevant allosteric residues. We also predicted the potential ligand binding sites and performed virtual screening against one of these binding sites to compare the lead molecules for free and aSyn-bound MMP1. Our results pave the way for substrate-dependent virtual screening guided by molecular insights that may enable inhibition of aggregation and degradation of aggregates.

## Results and discussion

### MMP1 dynamics on aSyn-induced aggregates show activity-dependent conformations and correlations

**Figure 1:**
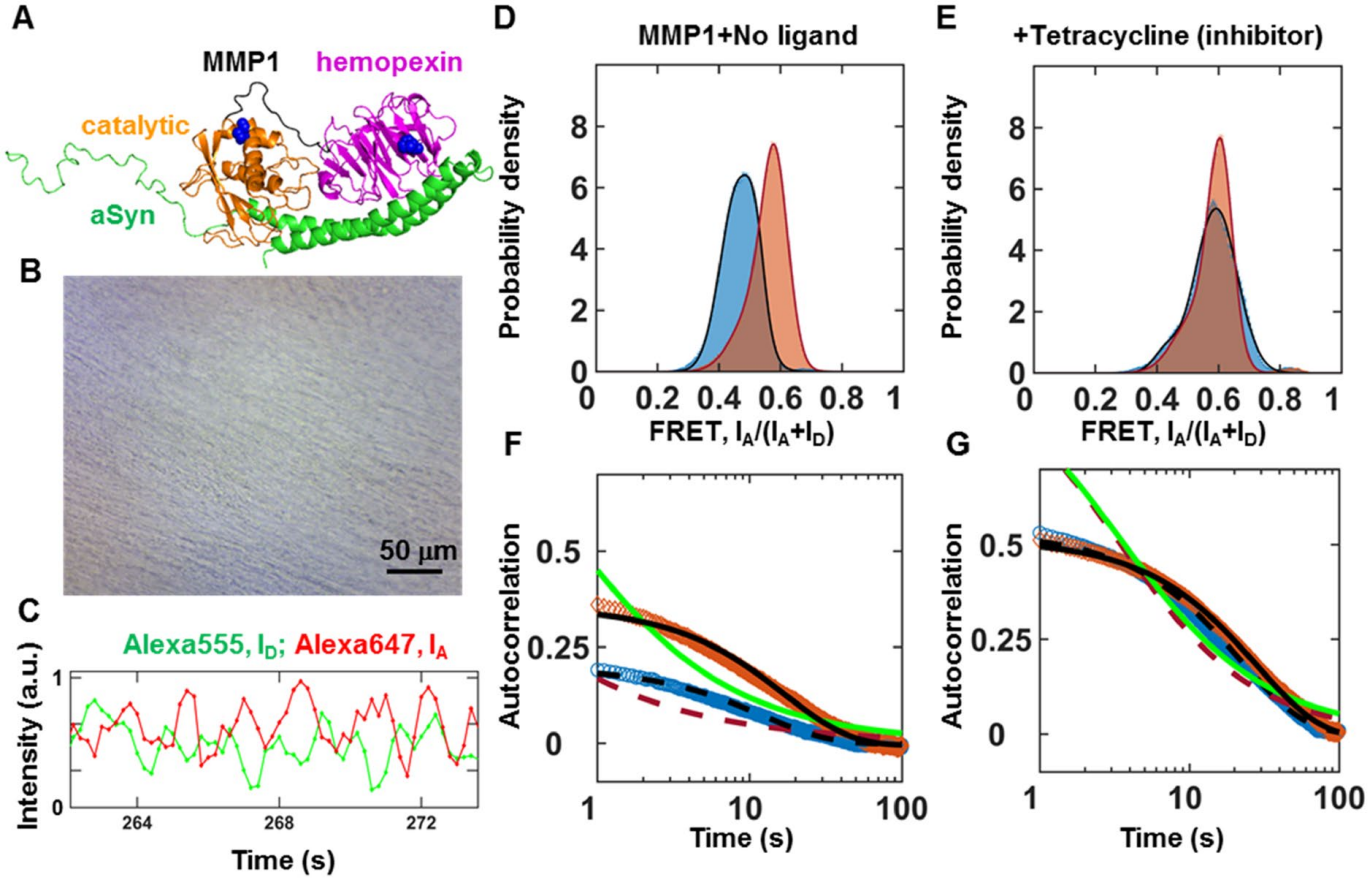
Activity-dependent interdomain dynamics of MMP1 on aSyn-induced aggregates at 22 ºC with 100 ms time resolution. (**A**) One of many predicted binding poses between MMP1 (PDB ID 4AUO; catalytic: orange; linker: black; hemopexin: magenta; S142C and S366C mutations in blue) and aSyn (PDB ID 1XQ8; green). (**B**) Light microscopy image of aSyn-induced aggregates on a slide. For MMP1-treated aggregates, see Fig. S1. For Transmission Electron Microscopy images of aggregates, see Fig. S2. (**C**) Emission intensities of the two dyes attached to active MMP1. (**D**,**E**) Area-normalized histograms of MMP1 interdomain distance (~ 200,000 FRET values, bin size = 0.005) without ligand and in the presence of tetracycline (an inhibitor), respectively, for active (blue) and inactive (orange) MMP1. All histograms are fitted to a sum of two Gaussians [Eq. (1) in methods] (active: solid blue line; inactive: solid red line). (**F**) and (**G**) Autocorrelations [Eq. (2) in methods] of MMP1 interdomain distance without ligand and in the presence of tetracycline, respectively, for active (blue) and inactive (orange) MMP1. All autocorrelations are fitted to exponentials and power laws [Eq. (3) in methods] (exponential fit to active: dashed black line; power law fit to active: dashed red line; exponential fit to inactive: solid black line; power law fit to inactive: solid green line). The error bars in histograms and autocorrelations represent the square roots of the bin counts and the standard errors of the mean (SEM) and are too small to be seen. For best-fit parameters, see Table S1.

Figure 1A shows one of many binding poses between MMP1 and aSyn predicted by ClusPro^51^. We purified catalytically active (E219) and inactive (Q219) MMP1 using a protease-based method described in our previous publication^52^. Both active and inactive MMP1 had the SER142CYS mutation in the catalytic domain and SER366CYS mutation in the hemopexin domain for labeling with the Alexa555 (donor)-Alexa647 (acceptor) FRET pair. Labeling does not affect the activity of MMP1^36^. To distinguish the effects of enzymatic activity despite potential problems due to dyes' photophysical properties, we used the inactive MMP1 as a control. Since aSyn-induced aggregates are water-insoluble, we created a thin layer of aggregates on a quartz slide (Fig. 1B). We flowed water-soluble MMPs to image the dynamics of MMP1 using a Total Internal Reflection Fluorescence (TIRF) microscope. We used a laser at 532 nm to excite Alexa555 (see methods). MMP1 undergoes interdomain dynamics and the distance between Alexa555 and Alexa647 changes. As a result, the non-radiative energy transfer due to FRET between the two dyes changes. Low FRET conformations lead to high Alexa555 emission, whereas high FRET conformations lead to low Alexa555 emission. Anticorrelated Alexa647 and Alexa555 emissions, I_A_ and I_D_, respectively, indicate the conformational dynamics of MMP1 (Fig. 1C). We calculated FRET using the equation I_A_/(I_A_ + I_D_), where each FRET value determines the separation between the two MMP1 domains. We plotted the distribution of FRET values to assess MMP1 conformations on aSyn aggregates (Fig. 1D). Active MMP1 prefers low FRET values in comparison to inactive MMP1, which suggests that MMP1 adopts open conformations on aSyn aggregates, similar to MMP1 conformations on collagen fibrils^36^ and fibrin^37^. In the presence of tetracycline, an MMP1 inhibitor^27^, active and inactive MMP1 adopt similar conformations (Fig. 1E), suggesting that tetracycline likely binds MMP1 to inhibit interdomain dynamics^36^. We calculated the correlation [Eq. (2) in methods] between conformations to determine how a conformation at one time is related to another conformation at a later time (Fig. 1F,G). We fitted both power law and exponential distributions to the autocorrelations because these are the most common decay types of correlations. MMP1 dynamics have correlations on aSyn aggregates, and an exponential fits the correlations, which led to the identification of allosteric residues in MMP1 having strong correlations with the catalytic motif residues of MMP1 (see later).

### A two-state Poisson process describes MMP1 dynamics on aSyn-induced aggregates

Recently, we published a quantitative analysis of MMP1 dynamics on collagen fibrils^36^ and fibrin^37^. The histograms reveal conformations of MMP1, and the autocorrelations show the relation between conformations at different time points. A sum of two Gaussians [Eq. (1) in Methods] fits the histograms of smFRET values, and an exponential fits the autocorrelations [Eq. (3) in methods]. As such, a two-state Poisson process describes the conformational dynamics of MMP1 and enables a straightforward interpretation of the decay rates of correlations^36^. We can establish a quantitative connection between the two states obtained from the Gaussian fits and the exponential fits' decay rates. We defined the two Gaussian centers as the two states, S1 (low FRET value) and S2 (high FRET value). Also, we described the two kinetic rates as k1 (S1S2) and k2 (S2S1) for interconversion between S1 and S2. For a two-state system, the ratio of rates (k1/k2) is the ratio of Gaussian area(S2)/area(S1), and the sum of rates (k1 + k2) is the decay rate of autocorrelation. We calculated k1 and k2 from k1/k2 and k1 + k2 for both active and inactive MMP1. Using experimentally determined S1, S2, k1, k2, and noise (widths of the histograms), we simulated smFRET trajectories and analyzed the simulated data similar to the experimental data. Comparing experimentally determined inputs (Table S1) and recovered parameters from two-state simulations (Table S2) shows that a two-state Poisson process describes MMP1 interdomain dynamics.

The two states are S1 = 0.46 and S2 = 0.52 on aSyn-induced aggregates for active MMP1 without ligand (Table S1). In comparison, the two states are S1 = 0.44 and S2 = 0.55 on collagen^36^ and S1 = 0.42 and S2 = 0.51 on fibrin^37^ for active MMP1 without ligand. The correlation decay rate of 0.08 s^−1^ on aSyn-induced aggregates for active MMP1 without ligand (Table S1). In comparison, the decay rates are 0.13 s^−1^ on collagen^36^ and 0.08 s^−1^ on fibrin^37^ for active MMP1 without ligand. In the presence of tetracycline, the two-state description still applies even though the two states and interconversion rates between them change (Table S1).

**Figure 2:**
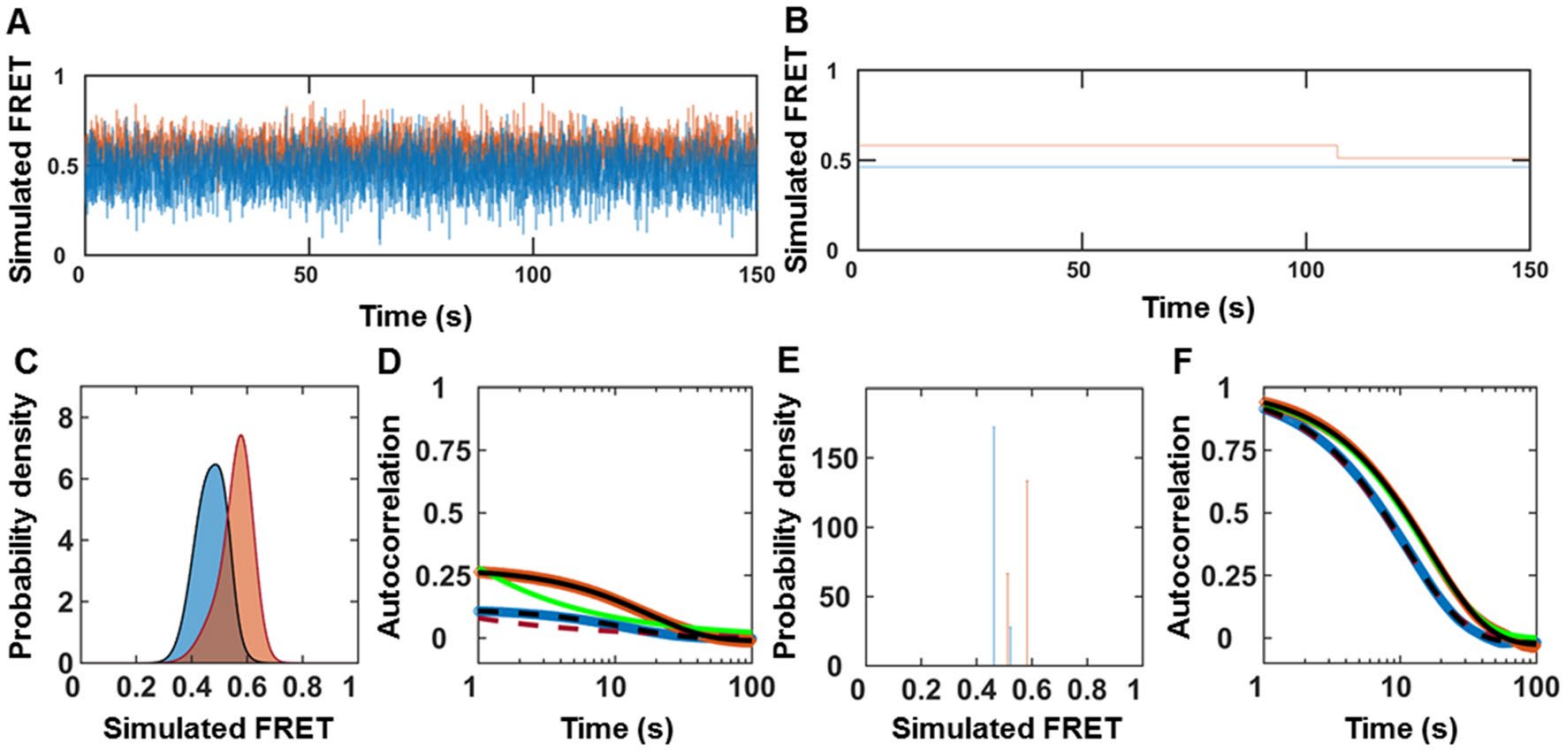
Stochastic simulation of MMP1 interdomain dynamics as a two-state system. (**A**,**B**) Examples of simulated smFRET trajectories with and without noise, respectively, for active MMP1 (blue) and inactive MMP1 (orange) using experimentally determined parameters for MMP1 without ligands. (**C**) Area-normalized histograms of simulated smFRET values with noise (active: blue; inactive: orange) with best fits to a sum of two Gaussians (solid line). (**D**) Autocorrelations of simulated smFRET trajectories with noise (active: blue; inactive: orange) with fits to exponentials (active: dashed black line; inactive: solid black line). Power law does not fit autocorrelations (active: dashed red line; inactive: solid green line). (**E**) Area-normalized histograms of simulated smFRET values without noise (active: blue; inactive: orange). (**F**) Autocorrelations of simulated smFRET trajectories without noise (active: blue; inactive: orange) with fits to exponentials (active: dashed black line; inactive: solid black line). Both exponential and power law fit autocorrelations (active: dashed red line; inactive: solid green line). The error bars are the SEMs for histograms and autocorrelations and are too small to be seen. For best-fit parameters, see Table S2.

We performed two-state simulations with and without noise to study how noise affects the histograms and autocorrelations (Fig. 2A–F). Only an exponential fits the autocorrelations with noise (Fig. 2D). However, both power law and exponential fit the autocorrelations of simulated smFRET trajectories without noise (Fig. 2F). In other words, the presence of noise can convert a power law correlation into an exponential correlation. This effect is similar to converting a Lorentzian line shape into a Gaussian line shape^53^. An exponential fit recovers the simulated kinetic rates' underlying sum with and without noise (Table S2).

### MMP1 dynamics depend on the aSyn-MMP1 binding pose

**Figure 3:**
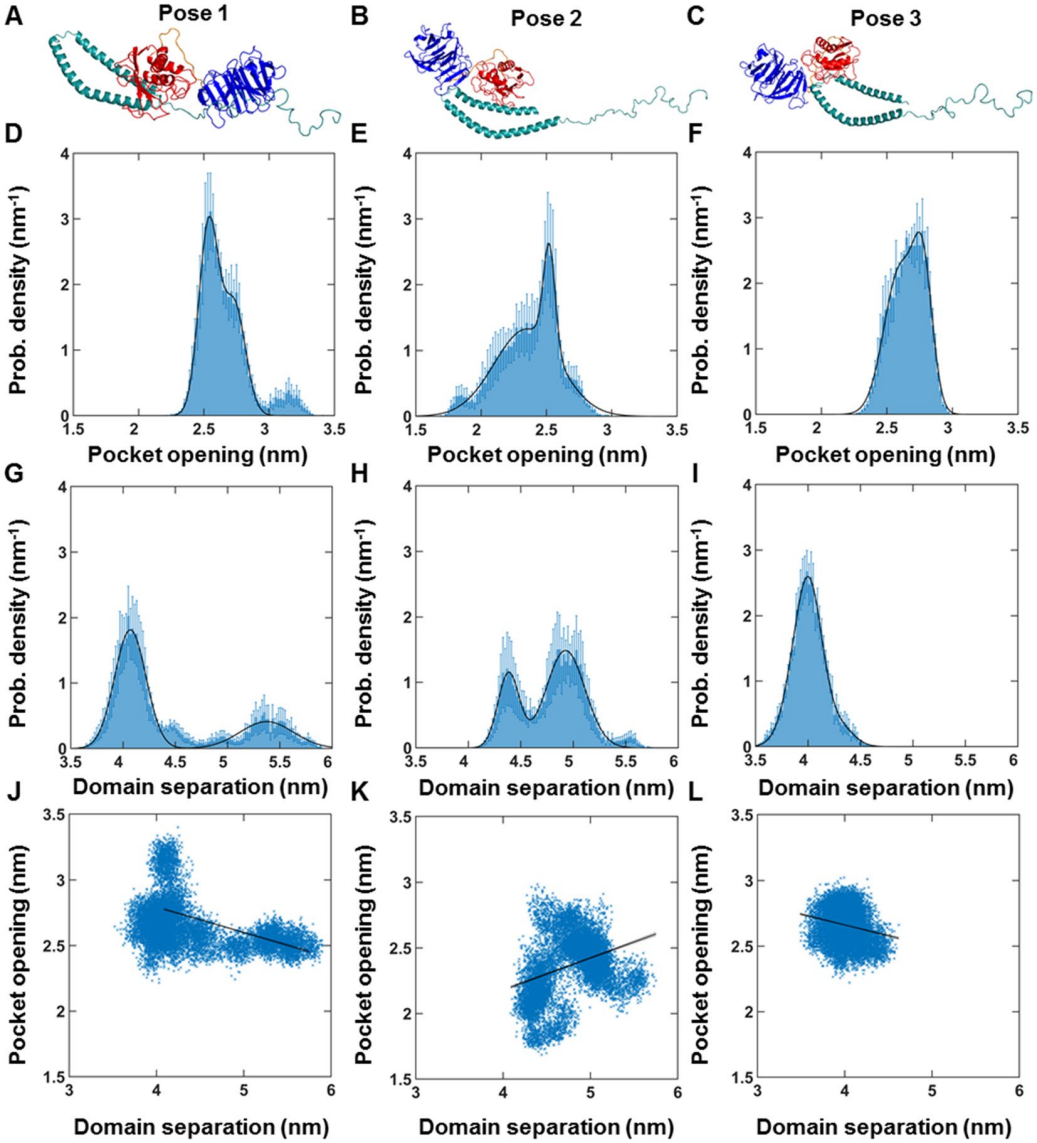
MMP1-aSyn binding pose-dependent dynamics of active MMP1 at 37 °C. (**A**,**B**,**C**) Three binding poses of MMP1 (the catalytic domain in red) at different locations of aSyn. (**D**,**E**,**F**) Area-normalized histograms of the catalytic pocket opening (N171-T230 distance) of MMP1 for poses 1, 2, and 3, respectively. (**G**,**H**,**I**) Area-normalized histograms of interdomain separation of MMP1 (S142-S366 distance) for poses 1, 2, and 3, respectively. (**J**,**K**,**L**) Linear correlation plots of catalytic pocket opening and interdomain distance for poses 1, 2, and 3, respectively. The data were fitted to $y_{i} = b_{0} + \,b_{1} \times x_{i}$[Eq. (4) in methods]. Note that a larger domain separation corresponds to a lower FRET value. Time resolution = 2 fs; Data saved every 5 ps; RMSD stabilization time for MMP1 =  ~ 5 ns; Total simulation duration = 20 ns. For calculations of linear correlations, see methods. For best-fit parameters, see Table S3. The error bars in Fig. 3D–I represent the standard deviations of three repeats of simulations for each condition. Figure 3J–L show combined data from the three repeats.

Since crystal structures of aSyn-bound MMP1 do not exist, we predicted the binding poses of aSyn (PDB ID 1XQ8) and MMP1 (PDB ID 4AUO) computationally using molecular docking software ClusPro^51,54^. Figure 3A–C show three such binding poses. MMP1 cleaves aSyn and produces several fragments, including aSyn fragments with amino acids M1-K21, M1-G41, M1-G47, M1-Y133, M1-Q134, L8-A140, A19-A140, V71-A140, T72-A140, Q79-A140, A91-A140, and D98-A140^13^. We selected three poses based on these cleavage fragments of aSyn near amino acids K45 (pose 3), T75 (pose 2), and A91 (pose 1). We leveraged known cleavage sites of MMP1 because the computational binding energy may not be the best indicator of appropriate docking poses^55^. We performed all-atom MD simulations for the three binding poses (Fig. 3) and calculated the catalytic pocket opening (Fig. 3D–F) and interdomain separation (Fig. 3G–I) of MMP1. Since MMP1 stabilized within ~ 5 ns (Fig. S3), we performed simulations for 20 ns. We chose pose 3 for further investigation because the distribution of interdomain distance (Fig. 3I) shows a similarity with the experimental distribution (Fig. 1D).

Additionally, we performed experiments on aSyn aggregates, and aggregation occurs due to C-terminal fragmentation, and as such, pose 3 presents a vulnerable position for MMP1 activity on aggregates. Aggregation may also reduce the intrinsic disorder of aSyn by forming structured fibrils^56^. Nevertheless, there are two important caveats. First, we performed simulations on aSyn monomer but performed experiments on aggregates. We took a similar approach for collagen and found that simulations on collagen monomer agreed with experiments on collagen fibril when we restrained the collagen backbone^36^, suggesting strains in collagen monomers inside collagen fibrils. For aSyn, we did not have to restrain the aSyn backbone for agreement between simulations and experiments, suggesting a lack of strain in aSyn-induced aggregates. Second, aSyn is considered an intrinsically disordered protein^57^ that assumes a structure after binding substrate^58^ or forming aggregates. Also, aSyn purified from neuronal and non-neuronal cell lines generally suggests a "natively folded" ~ 58 kDa tetrameric form^59,60^. Nevertheless, the combined experiment-simulation approach using the monomer structure of aSyn provides a starting point for molecular-level understanding.

### Shannon entropy enables the quantification of allosteric communications

**Figure 4:**
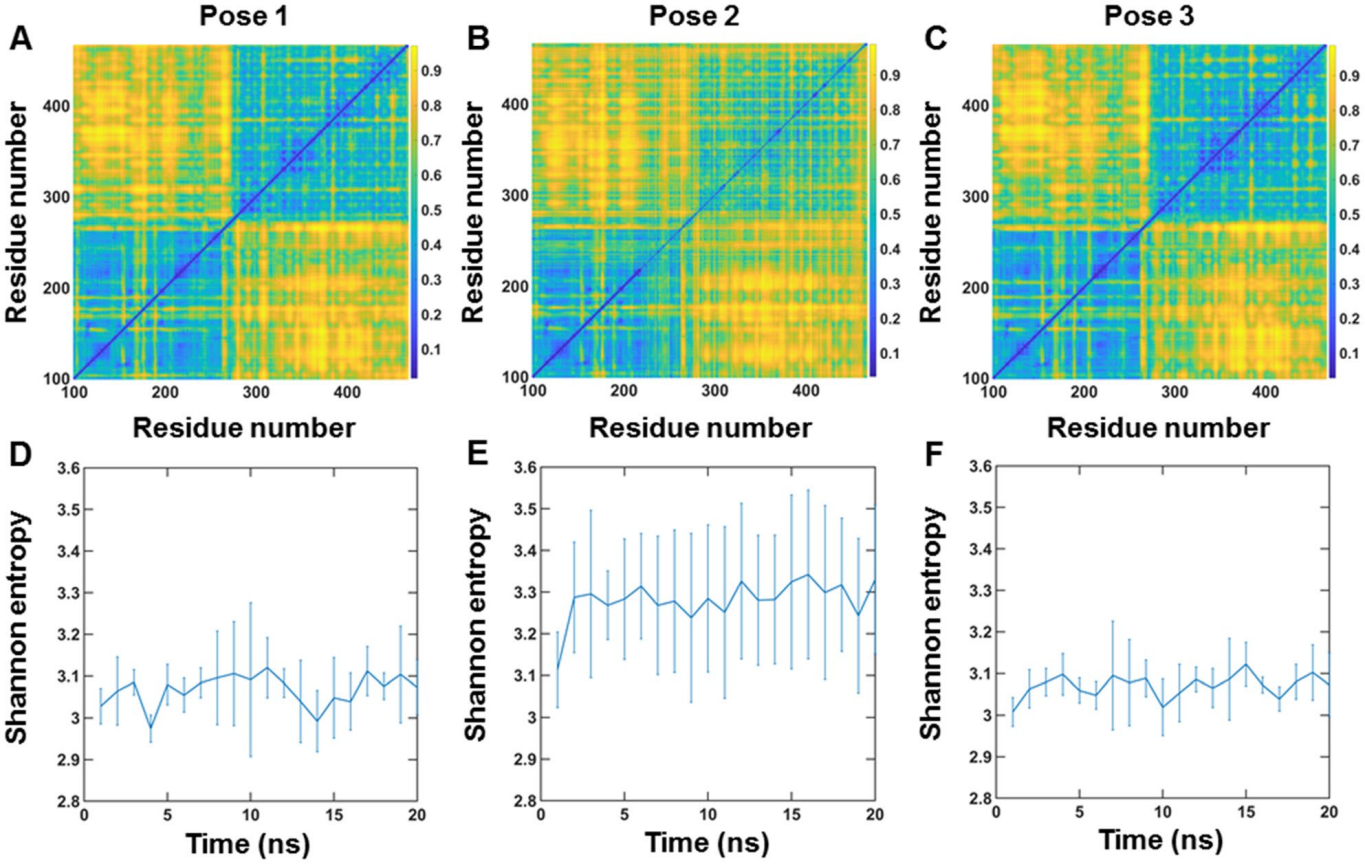
Pose-dependent correlation between residues in active MMP1 and quantification of allostery using Shannon entropy at 37 °C. (**A**,**B**,**C)** Mean correlations between residues in three repeats of simulations for poses 1, 2, and 3, respectively, at 37 °C. Correlations are normalized between 0 (blue) and 1 (yellow). Yellowish colors indicate higher correlations. (**D**,**E**,**F**) Shannon entropy calculated from correlation plots for poses 1 (S = 3.07 ± 0.04, mean ± SEM), 2 (S = 3.28 ± 0.05, mean ± SEM), and 3 (S = 3.07 ± 0.03, mean ± SEM), respectively. For details, see supplementary information and Fig. S4. The error bars in (**D**–**F**) represent the standard deviations of three repeats of simulations for poses 1, 2, and 3.

Since a correlated motion suggests a decrease in randomness or lower entropy, we calculated correlations between each pair of residues in MMP1 and estimated entropy (Fig. 4). We divided all-atom simulations into 1 ns long windows. In each 1 ns window, there were 200 radial coordinates for residues. We calculated correlations of fluctuations [Eq. (2) in methods] and normalized it to a range of 0 to 1 by subtracting the minimum and then dividing by the maximum. Figure 4A–C show the matrix of correlation values at lag number 1, averaged over 20 ns (see supplementary information for the meaning of lag numbers and correlation calculations). We divided the correlation values to create 10 bins of width 0.1, calculated 10 × 10 Gy-level co-occurrence matrix (GLCM), and defined Shannon entropy $S = - \sum {p_{i} \ln } p_{i}$, where $p_{i}$ is the probability of a microstate $i$. The catalytic domain residues (F100-Y260) have strong correlations with the hemopexin domain residues (D279-C466), suggesting allosteric communications in MMP1 (Fig. 4A–C). The time-evolutions of Shannon entropy are shown in Fig. 4D–F. Pose 3 has the lowest entropy consistent with the functionally relevant binding pose.

**Figure 5:**
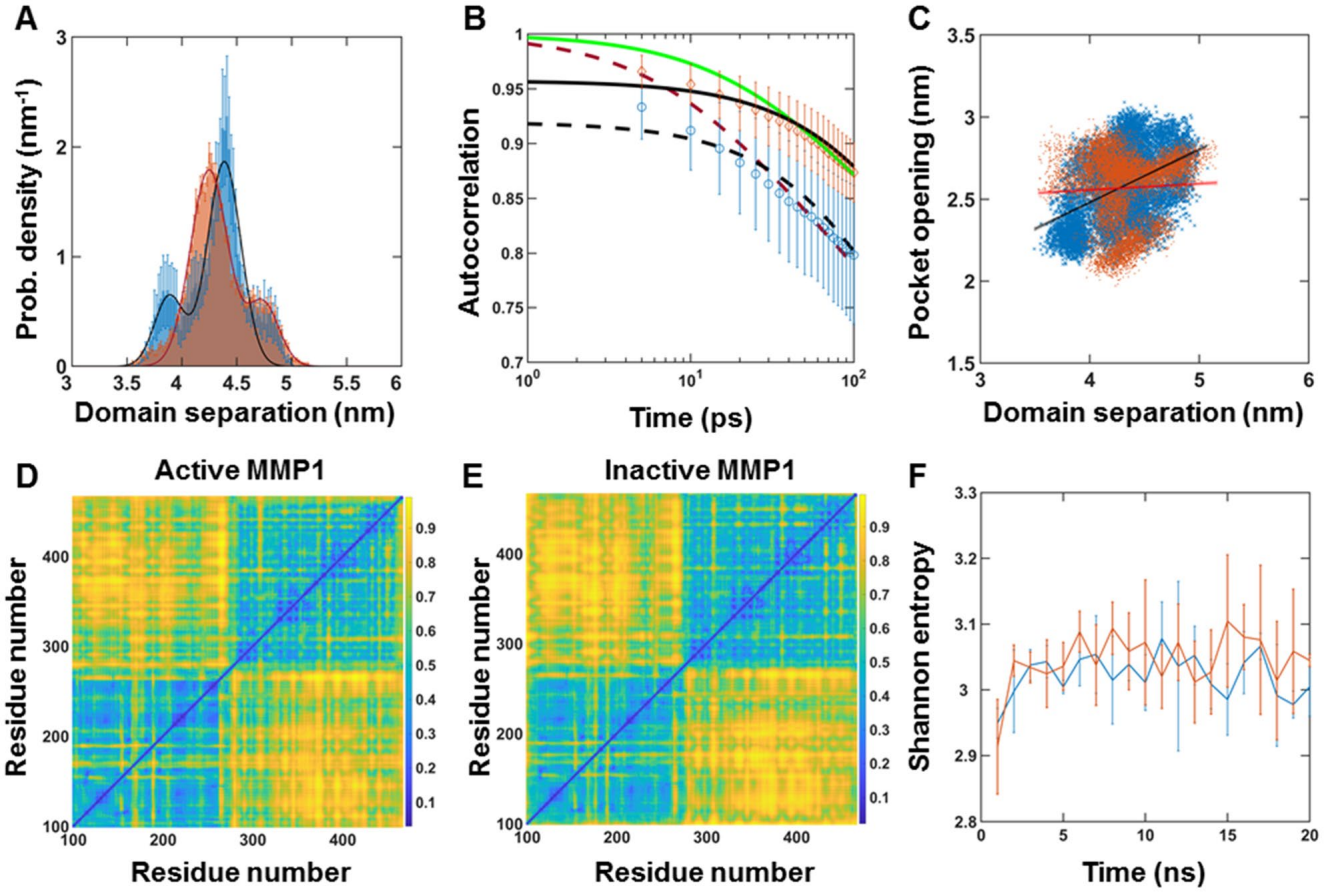
All-atom MD simulation of MMP1 interdomain dynamics of pose 3 at 22 °C. (**A**) Area-normalized histograms of simulated interdomain distance (active: blue; inactive: orange) with best fits to a sum of two Gaussians (solid line). (**B**) Autocorrelations of simulated interdomain distance (active: blue; inactive: orange) with fits to exponentials (active: dashed black line; inactive: solid black line). Power law does not fit autocorrelations (active: dashed red line; inactive: solid green line). (**C**) Linear correlation plots of catalytic pocket opening and interdomain distance. The plot shows combined data from the three repeats of simulations for pose 3. (**D,E**) Mean correlations between residues of three repeats of simulations for pose 3 for active and inactive MMP1, respectively. Correlations are normalized between 0 (blue) and 1 (yellow). Yellowish colors indicate higher correlations. (**F**) Shannon entropy calculated from correlation plots for active (S = 3.02 ± 0.03, mean ± SEM) and inactive (S = 3.04 ± 0.04, mean ± SEM). For best-fit parameters, see Table S4. The error bars in (**A**,**B**,**F**) represent the standard deviations of three repeats of simulations for pose 3. (**C**) shows combined data from the three repeats.

We performed MD simulations of pose 3 at 22 °C to compare with smFRET measurements at 22 °C. Figure 5A shows the distributions of the interdomain distance between S142 and S366. In agreement with experiments (Fig. 1D), active MMP1 adopts conformations with the two domains separated more than inactive MMP1. Also, simulated dynamics correlations (Fig. 5B) follow the experimental pattern (Fig. 1F), with inactive MMP1 having higher values with longer correlation times. We used experimentally consistent simulations to gain further insights. First, the correlation between the catalytic pocket opening and interdomain separation for active MMP1 is higher than inactive MMP1 (Fig. 5C). A larger catalytic pocket opening enables substrates to get closer to the active site. Second, the two-dimensional correlation plots show allosteric communications between the two domains (Fig. 5D,E). Third, Shannon entropy describing the randomness of two-dimensional correlations plots shows a lower value for active MMP1 than inactive MMP1 (Fig. 5F).

We also repeated simulations of pose 3 at 22 °C with two zinc and four calcium ions in MMP1 (Fig. S5). Two main insights remain the same with and without ions. First, active MMP1 prefers an open conformation (more separation between domains or low FRET), which is consistent with experiments (Fig. 1D), 100 ns simulations with ions (Fig. S5A), and 20 ns simulations without ions (Fig. 5A). Second, MMP1 has strong allosteric communications between domains, which is consistent with 100 ns simulations with ions (Fig. S4D,E) and 20 ns simulations without ions (Fig. 5D,E). Although ions, especially the catalytic Zn, are critical for the final hydrolysis step by MMPs, they may be less influential on structural dynamics^61^.

Since we obtained similar insights with and without metal ions, we performed all other simulations without metal ions. Without metal ions, validated force field parameters for the 20 natural amino acids are already available and can be used for molecular dynamics simulations of any protein with a structure. However, the force field parameters for metal ions must be calculated for each case because these parameters depend upon the nature of the metal ion, its coordination number, and its geometrical arrangement^62^. As such, molecular dynamics simulations with metal ions are more complicated and time-intensive, and we performed simulations without metal ions.

### Changes at the catalytic site and identification of allosteric residues in MMP1

**Figure 6:**
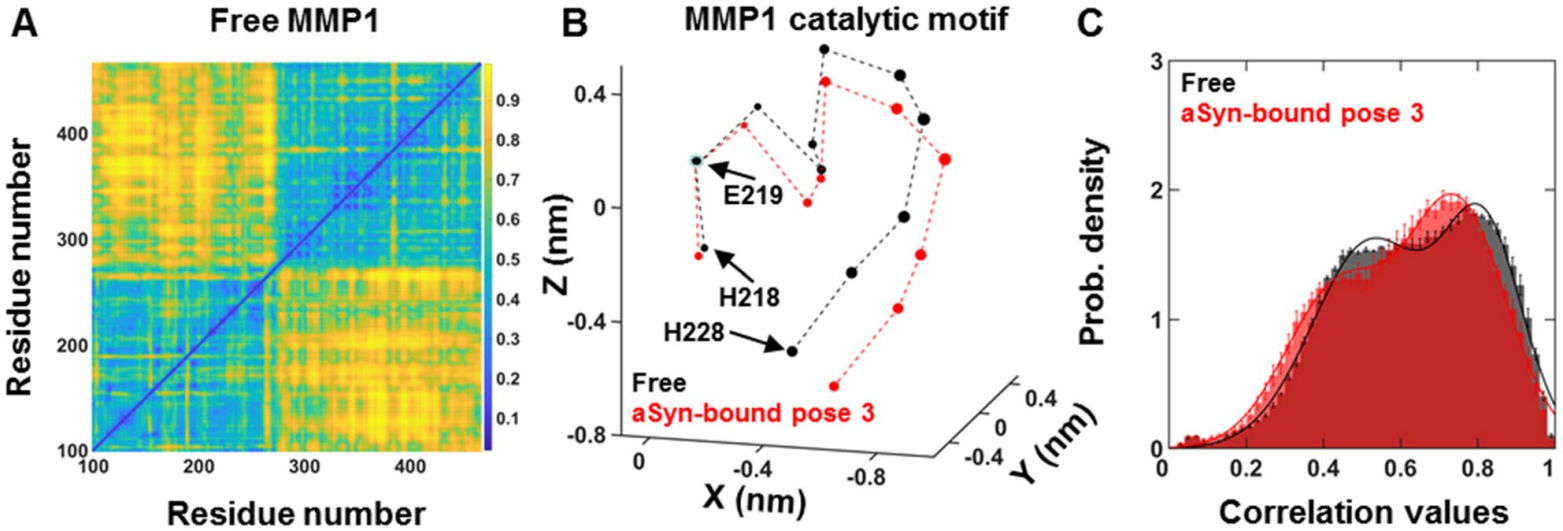
Conformational changes near the catalytic motif and identification of allosteric residues in MMP1 at 37 °C. (**A**) Normalized correlations between each pair of residues for free active MMP1 at 37 °C. (**B**) Three-dimensional configurations of the catalytic motif residues for free active MMP1 (black) and aSyn-bound MMP1 (red) at 37 °C. (**C**) Histograms of correlation values of free MMP1 (black) and aSyn-bound MMP1 (red). For best-fit parameters, see Table S6.

We performed MD simulations for free MMP1 (Fig. 6A) and compared them with the simulations (Fig. 4C) for pose 3 (Fig. 3C) of aSyn-bound MMP1. The comparison revealed how the conformations of MMP1 catalytic motif HELGHSLGLSH changed. We considered the catalytic residue E219 as the origin, calculated the average (x,y,z) coordinates of all atoms in each amino acid to define the amino acid's location, and plotted the pairwise distance with the catalytic motif residues in three dimensions. The symbol size of the locations is proportional to the standard deviation of the pairwise distance (Fig. 6B and Fig. S6). The configuration at the MMP1 catalytic site changes considerably as free MMP1 binds aSyn.

We plotted histograms of correlation values for free and aSyn-bound MMP1 and determined the threshold correlation values at 0.8 (peak probability density ~ 2.2 divided by 2.7) (Fig. 6C). We found all the residues having normalized correlations greater than 0.8 in Figs. 4C, 6A with the catalytic motif residues HELGHSLGLSH and compared the residues between free MMP1 and aSyn-bound MMP1. We identified residues in the hemopexin domain between D279 and C466 (although full-length MMP1 has 469 residues, PDB ID 4AUO has residues till C466) having exclusive correlations with the catalytic motif residues only MMP1 binds aSyn. These residues, based on three repeats of simulations, are K281, T283, G292, G327, L328, E329, R337, F343, G345, N346, Y348, G353, Q354, D363, Y365, S366, S367, F368, P371, R372, V374, K375, A379, F391, A394, R399, M414, F419, V426, and C466. Due to the inherent randomness of protein dynamics, there are variabilities in identified residues. Figure S7 shows these allosteric residues in MMP1, some of which are surface exposed and, as such, can be targeted for virtual screening. Identifying allosteric residues is significant because it may be possible to identify exclusive residues for other substrates of MMP1 and control one MMP1 function without affecting the other functions.

### Lead molecules from virtual screening against MMP1 depend on the binding pose

**Figure 7:**
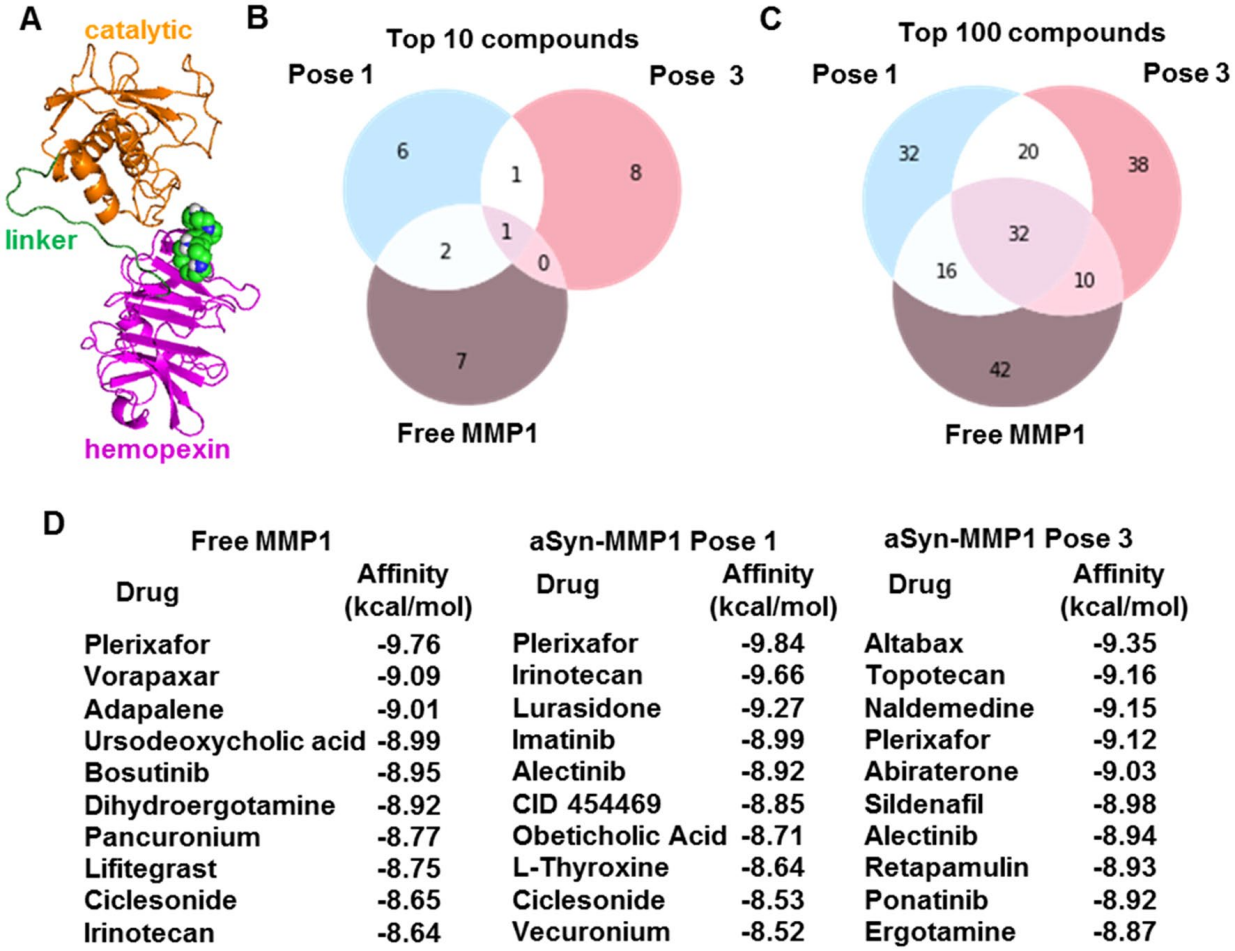
Substrate- and pose-dependent virtual screening against MMP1. (**A**) MMP1 bound to Plerixafor (green and blue spheres), the top hit against free MMP1. We performed virtual screening against the same site for free MMP1, pose 1, and pose 3. (**B,C**) Venn diagrams of top 10 and 100 molecules show unique and common ligands. (**D**) Identities and affinities for the top 10 molecules. For docking parameters, see supplementary information.

Guided by experiments and simulations, we determined that pose 3 is likely a relevant binding pose between MMP1 and aSyn. Therefore, we used the experimentally-informed binding pose for the virtual screening of molecules. Virtual screening enables testing considerably more ligands economically and faster than high-throughput experimental screening. A wide range of strategies can be divided into ligand-based and structure-based virtual screening^63^. In ligand-based virtual screening, known ligands against a target serve as benchmarks to screen for more ligands with similar properties. In contrast, structure-based screening uses the binding sites on a target to screen molecules leveraging the conformational changes and energetic complementarity upon ligand binding. Since MMP1 has broad-spectrum protease activity, we determined the binding sites on free MMP1 and aSyn-bound MMP1 to investigate a substrate's effects. We predicted the potential ligand-binding sites using AutoLigand and MGLTools 1.5.7 and selected a potential binding site in the hemopexin domain having significant overlap with the identified allosteric residues in the last paragraph (Fig. 7A).

We performed screening against the selected site in the hemopexin domain for free MMP1 and two poses of aSyn-bound MMP1. We performed molecular docking using AutoDock^64^ and set up the virtual screening with Raccoon 1.1. We used default AutoDock docking parameters (see supplementary information) to determine the binding affinities for a collection of 1400 FDA-approved compounds from the ZINC15 database^65^. We used FDA-approved molecules because those provide a smaller set of commercially available molecules. We fixed the otherwise flexible side chains. Flexible side chains would have given slightly more accurate screening but would require more computational time. We found that the lead molecules against MMP1 change depending on the substrate and pose. Figure 7B–D show the top molecules for free MMP1 and the two aSyn-MMP1 binding poses, suggesting that substrate binding and different poses can lead to different results. Venn diagrams of molecules (Fig. 7B,C) show that there are molecules unique to each case. The ligand-binding site in the hemopexin domain was common to free MMP1, pose 1, and pose 3. However, conformational differences in the three cases clearly impact virtual screening. The unique molecules for each case suggest that we should perform substrate-specific screening to identify unique ligands, thus resolving problems arising from MMP promiscuity.

### Substrate-specific allosteric residues in MMP1 enable single molecule insights into the selection of lead molecules

**Figure 8:**
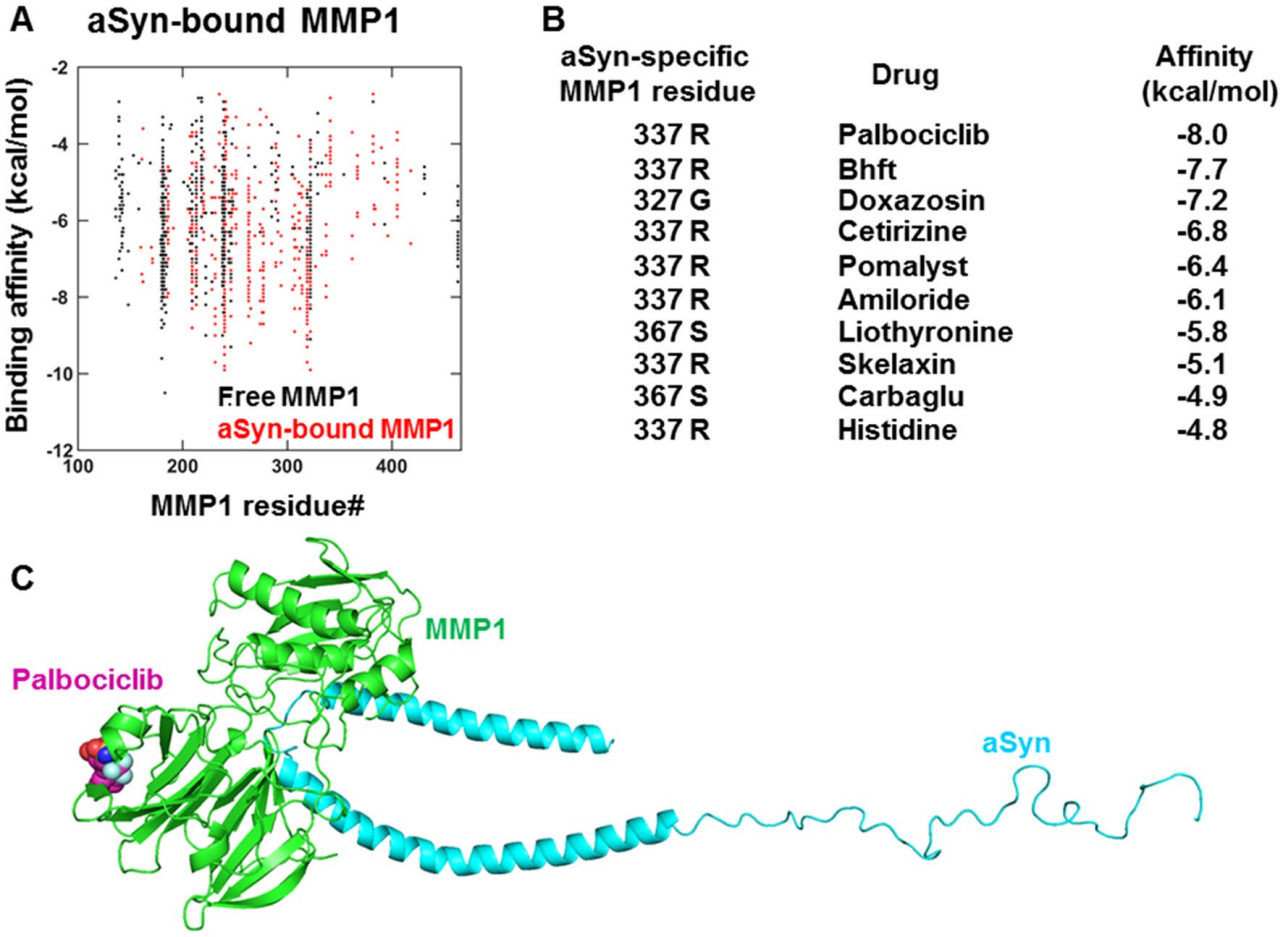
Selection of lead compounds based on substrate-dependent allosteric residues in MMP1. (**A**) Binding affinities of lead compounds and closest MMP1 residues for aSyn-bound MMP1. (**B**) Compounds that bind to aSyn-specific allosteric residues in MMP1. (**C**) Palbociclib bound to aSyn-bound MMP1. Lead molecules depend on whether MMP1 is free or bound to a substrate. Also, not all identified allosteric residues have associated lead molecules. Allosteric residues reduce the number of lead molecules significantly.

As a proof of principle, we wanted to check if aSyn-specific allosteric residues may help the selection of drugs because aSyn has its unique signature or "fingerprint" on MMP1 at the catalytic and distant allosteric sites. To this end, we screened ~ 9000 FDA-approved drugs (see methods) from the ZINC15 database and selected compounds for which the difference between the binding affinities at the top two binding modes is at least 10% because we want to find drugs that bind preferably to a specific site on MMP1. With a 10% difference, we found ~ 600 drugs out of ~ 9000 starting drugs. We considered the top affinity site for each compound, identified the MMP1 residue closest to each drug, and plotted it in 2D (Fig. 8A). Then, we selected drugs further by identifying drugs binding to the aSyn-specific allosteric residues (Fig. 8B), leading to only a few lead molecules. Figure 8C shows how Palbociclib binds to an aSyn-specific MMP1 residue. These results suggest that we may be able to identify drugs with an exclusive binding preference for an allosteric site on MMP1 by screening a larger number of compounds, potentially reducing off-target effects. Since MMPs interact with and degrade many biomolecules in the human body, substrate-specific allosteric residues or "allosteric fingerprints" may alter one MMP1 function without affecting its other activities using allosteric ligands.

## Conclusions

In summary, we measured the interdomain dynamics of MMP1 on aSyn-induced protein aggregates and modeled the dynamics as a two-state Poisson process. Distributions of conformations and correlation decay rates of MMP1 on aSyn-induced aggregates follow the general pattern that we previously reported for collagen^36^ and fibrin^37^, i.e., open MMP1 conformations are functionally relevant, and there are time-dependent correlations of conformations.

A comparison of experiments and simulations suggested that the pose in which MMP1 binds to aSyn near amino acid 45 (pose 3) leads to a better match with experiments. Simulations further suggested that the configuration of the catalytic motif HELGHSLGLSH differs significantly between free and aSyn-bound MMP1. We identified allosteric residues in the hemopexin domain between D279 and C466, having strong correlations with the catalytic motif residues. These allosteric residues in the hemopexin domain may enable engineering MMP1 activity due to their strong correlations with the catalytic motif.

We found that the potential ligand-binding sites change upon binding aSyn and depend on the binding pose. The lead molecules and their scores against the selected site having significant overlap with identified allosteric residues change upon binding the substrate and depend on the binding pose. Therefore, substrate-dependent binding sites and lead molecules suggest that any effort to target MMP1 may need to consider the substrate, the binding pose, and the ligand-binding site. In the future, the synergistic combination of experiments and simulations may enable modulation of MMP1 activity using molecular insights at the single molecule level. It may pave the way for substrate-specific control of MMP activity using allosteric ligands.

## Materials and methods

### Purification of MMP1 and aSyn

The MMP1 and aSyn sequences were inserted into the pET21b + and pET11a vectors, respectively, between NdeI (N-terminal) and HindIII (C-terminal) restriction sites. We transformed the plasmids into Rosetta (DE3) pLysS *E. coli* (Millipore, Cat# 70956-4) for protein expression. We purified MMP1 and aSyn, as described in our previous publications^52,66^. The method of purifying aSyn also produced aSyn-induced aggregates used in experiments.

### Measurements of MMP1 interdomain dynamics

For smFRET measurements of MMP1 interdomain dynamics, we mutated two serine residues in MMP1 at locations 142 and 366 to cysteines for labeling with Alexa555 and Alexa647 dyes. Previously, we showed that labeling does not affect MMP1 activity^36^. In addition, we introduced the E219Q mutation in the catalytic domain to create a catalytically inactive mutant of MMP1. We prepared aSyn aggregates, as described in our previous publication^66^. We spread aSyn-induced aggregates and made a thin layer on a quartz slide. We made a flow cell for single molecule experiments using a piece of double-sided adhesive tape sandwiched between the quartz slide and a glass coverslip. Labeled MMPs were flowed into the flow cell and excited at 532 nm wavelength using the evanescent wave created at the quartz slide and sample buffer interface in a TIRF microscope. We acquired two-channel movies to detect emissions from Alexa555 and Alexa647. Any relative motion between the two MMP1 domains would lead to a non-radiative transfer of energy from Alexa555 to Alexa647 due to FRET, increasing the emission from Alexa647 (I_A_) and simultaneously reducing the emission from Alexa555 (I_D_). We calculated FRET efficiency by I_A_/(I_A_ + I_D_)^67^. Single-molecule experiments and analyses have been described in our previous publications^68–71^.

### All-atom simulations without metal ions

We removed all zinc and calcium ions and the water molecules from PDB ID 4AUO and replaced the missing side-chain atoms using Chimera's rotamer tool. The PDB files for active and inactive MMP1 were created by replacing A219 with E219 and Q219, respectively, using Pymol's mutagenesis function. We used Gromacs 2019.6 with the Gromos96 43a1 force field to perform the MD simulations. We ignore the hydrogen atoms while creating the topology file. Each simulation ran 20 ns long, simulated at 2 fs/step, and sampled every 5 ps. Each MMP1 crystal was placed in a cubic box with 3D period boundary conditions and solvated with water (using a single point charge model (SPC)) and Na counter-ions to create a neutral system. We then used the steepest descent algorithm to minimize the solution's energy. To equilibrate the solution with the protein complex, we used NVT and NPT ensemble simulations. First, we performed the NVT simulation and set the mean temperature at the desired temperature of 295 K (22 °C) or 310 K (37 °C) using a Berendsen thermostat for 100 ps. We used the Verlet cut-off scheme for neighbor searching and updated the neighbor list every 20 fs. We used the particle mesh Ewald scheme to calculate the electrostatic interactions with a cubic interpolation order of 4 and a cut-off at 1 nm. We assigned initial velocities using a Maxwell distribution from the corresponding temperature. In the NPT simulation, we maintained velocities from the NVT simulation output, and the pressure was set to 1 bar using a Parrinello-Rahman barostat for an additional 100 ps. Once the NVT and NPT simulations were finished and the system was equilibrated, we removed the position restraints and ran the production MD for 20 ns. We edited the final coordinates in the trajectory file to correct for periodicity and center the protein complex. The distances between the catalytic pocket residues and the serine residues (interdomain distance) are then measured at every time step using the Gromacs distance function. We measured the interdomain separation between the alpha carbon atoms of residues S142 and S366 and the catalytic pocket opening between the alpha carbon atoms of residues N171 and T230. To calculate the correlation and Shannon entropy, we recorded the alpha carbon atom's coordinates in each residue of MMP1.

### Analysis of experimental and simulated interdomain dynamics

We chose the histograms' bin width to be at least the inverse of the sample size's square root, i.e., $1/\sqrt N$, N is the number of data points. The chosen bin size is closer to the bin size predicted by Scott's rule of bin size^72^. We calculated the error of count in each bin as the square root of the bin count. Both the bin counts and errors were divided by the histogram area to create the area-normalized histogram (probability density function). The area under the normalized histogram equals 1. We fitted a sum of two Gaussians to the histograms:

1 $$ y = a_{1} \times e^{{ - \frac{{\left( {x - b_{1} } \right)^{2} }}{{c_{1}^{2} }}}} + a_{2} \times e^{{ - \frac{{\left( {x - b_{2} } \right)^{2} }}{{c_{2}^{2} }}}} $$

where a's, b's, and c's are amplitudes, centers, and widths of the Gaussians. The parameters b1 and b2 are the two states, S1 and S2.

To calculate correlations, we subtracted the average value from each trajectory and used the following equation to calculate correlations:

2 $$ C_{\tau } = \frac{1}{N - \tau }\sum\limits_{t = 1}^{N - \tau } {\left\{ {I(t) - \frac{1}{N - \tau }\sum\limits_{{t^{\prime} = 1}}^{N - \tau } {I(t^{\prime})} } \right\}} \times \left\{ {I(t + \tau ) - \frac{1}{N - \tau }\sum\limits_{{t^{\prime} = 1 + \tau }}^{N} {I(t^{\prime})} } \right\} $$

where $C_{\tau }$ is the correlation at lag number $\tau$, $N$ is the number of points in a FRET trajectory, and $I(t)$ is the FRET value at $t.$ For autocorrelations, both factors in curly brackets were from the same time series. For cross-correlations, the two factors in curly brackets were from different time series.

We normalized correlations by dividing correlation values at each lag by $C_{\tau = 0} .$ We fitted correlations between $\tau = 1$ and $\tau = 1000$ to both power law and exponential distributions. For power law, we used a form of Pareto distribution^73^ that satisfies the boundary conditions, i.e., $C_{\tau = 0} = 1$ at $t = 0$ and $C_{\tau = \infty } = 0$ at $t = \infty .$

We fitted the following equations of power law and exponential functions:

3 $$ \begin{aligned} C_{\tau } & = \left( {a \times \tau + 1} \right)^{ - b} \\ C_{\tau } & = d \times \exp^{ - e \times \tau } + f \\ \end{aligned} $$

To quantify linear correlations between the catalytic pocket opening and interdomain separation from simulations, we used the interdomain distance as the single predictor variable for the catalytic pocket distance in a linear model. A linear model describes a response variable as a function of predictor variables. Linear models are often fit using a method known as statistical regression. There are different regression methods used, depending on the number of predictor variables. When using only one predictor variable, the regression method is known as the simple linear regression, which we used for quantifying correlations. We used the following equation:

4 $$ y_{i} = b_{0} + \,b_{1} \times x_{i} $$

where *b*_*0*_ and *b*_*1*_ are the estimated fit parameters. There are also a few different methods to estimate the parameters of the linear model. The most-used approach is the least-squares operator, which finds the slope through the data that minimizes the squared distance between the fit and the residues. There is still uncertainty in these estimations, no matter which method we used. Confidence bands visually represent the uncertainty in linear models by showing the range of possible slopes. We calculated the 95% confidence interval of each predictor value's mean.

### Small molecule putative binding sites on MMP1

For virtual screening, we selected free MMP1 and two poses of aSyn-MMP1 binding. First, we predicted potential putative binding sites using AutoLigand and MGLTools 1.5.7. Next, we assigned hydrogen atoms (including polar hydrogens) and Gasteiger partial charges to each of the two aSyn-bound MMP1 poses and free MMP1. Zinc and calcium ions were removed. A grid map with 1 Å spacing was then applied to the hemopexin and catalytic domains in addition to the linker region, making the docking unbiased for different binding sites, especially allosteric ones. AutoLigand was then used to define four binding site calculations per structure, beginning with 100 points to define the volume of the binding envelope, then increasing the number of points to 200, 300, and finally 400. The binding envelopes defined were analyzed using AutoDock, and results were visualized using MGLTools and VMD.

### Pose-dependent virtual screening of small molecules against a putative site on MMP1

We performed molecular docking using AutoDock, and the virtual screening was set up with Raccoon 1.1. The receptor was considered rigid, and rotatable bonds imparted flexibility to the ligands. We used default AutoDock docking parameters (see supplementary information) to determine the small-molecule binding affinities. A collection of 1400 FDA-approved compounds were obtained from the ZINC15 database. We began by importing the ligand multiple structure MOL2 file into Raccoon. Each ligand was automatically converted into a separate PDBQT while settling hydrogen atoms and partial charges. We calculated a set of new grid map files with a grid spacing of 0.37 Å to include the selected putative binding site from the binding site prediction. For each of the three cases (free MMP1 and two aSyn-MMP1 poses), 100 ligands with the lowest binding affinities were identified. The lead molecules' ZINC IDs were converted to chemical IDs (CIDs) for comparison. The top 10 ligands were identified by their common names.

### Single molecule insights into the virtual screening

We used free MMP1 and binding pose 3 of aSyn-bound MMP1 as the receptors. We opened PDB files of MMP1 in AutoDockTools, added polar hydrogens, and created a .pdbqt file for the three receptors, i.e., free MMP1 and aSyn-bound MMP1. We selected ~ 9000 FDA-approved compounds from the ZINC15 database and downloaded the selected compounds as a .sdf file for ligands. We used OpenBabel to convert the .sdf file into a .pdbqt file, then AutoDock Vina to create individual .pdbqt files using vina_split on a Linux terminal. We also made a .txt file containing filenames for the compounds. We created a configuration file to set the parameters of screening in AutoDock Vina. The configuration file is a .txt file containing receptors' filename, docking box coordinates, number of modes, energy range, and exhaustiveness. We used the AutoDockTools GUI to obtain the coordinates by opening the Grid menu and selecting Grid Box. We adjusted the box to enclose the whole MMP1 to identify the binding poses of each compound and used exhaustiveness of 10 for quicker screening. After the virtual screening, we obtained two types of files: a .pdbqt file of the top 10 docking modes and a log of their binding affinity scores. We analyzed the results in Matlab.

## Supplementary Information

Supplementary Information.
