# Supplementary material for: Identification of allosteric fingerprints of alpha-synuclein aggregates in matrix metalloprotease-1 and substrate-specific virtual screening with single molecule insights
Source: Sci Rep. 2022 Apr 6;12:5764. doi: 10.1038/s41598-022-09866-7 (PMC8987064; doi:10.1038/s41598-022-09866-7)
Supplement: Supplementary file 1 — Supplementary Information. [file 41598_2022_9866_MOESM1_ESM.pdf]

Supplementary information

Identification of allosteric fingerprints of alpha-synuclein aggregates in matrix metalloprotease-1 and substrate-specific virtual screening with single molecule insights

Sumaer Kamboj[1,#], Chase Harms[1,#], Derek Wright[1], Anthony Nash[2], Lokender Kumar[1], Judith Klein-Seetharaman[3], and Susanta K. Sarkar[1,*]

[1]Department of Physics, Colorado School of Mines, Golden, CO, USA
[2]Nuffield Department of Clinical Neurosciences, University of Oxford, UK
[3]Department of Chemistry, Colorado School of Mines, Golden, CO, USA
[#]These authors contributed equally

Corresponding author:
[*]ssarkar@mines.edu

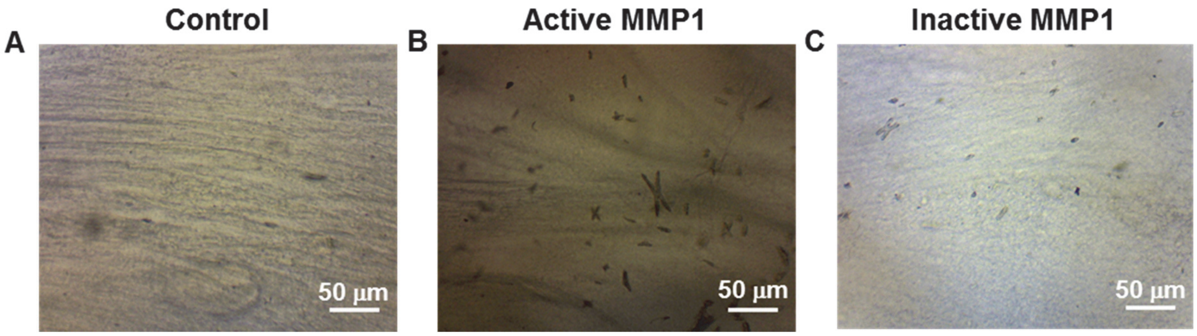

Figure S1. Light microscope images of aSyn-induced aggregates stained with Congo red. (**A**) Aggregates on a slide treated with protein buffer. (**B**) Aggregates on a slide treated with active MMP1 at 22 °C for 30 min. (**C**) Aggregates on a slide treated with inactive MMP1 at 22 °C for 30 min.

Transmission Electron Microscope (TEM) images of aSyn-induced aggregates. We used freshly glow-discharged 200-mesh formvar carbon-coated copper grids (Electron Microscopy Sciences, Cat# FCF200-CU-50). We dipped two wooden tips in aSyn-induced aggregates and separated the two tips to create a thin layer of aSyn-induced aggregates on the TEM grid. After drying the sample for 5 min, we soaked the sample with 5 μL of 2% uranyl acetate for 3 min and blotted any excess solution with a piece of Whatman filter paper. We rinsed the sample with 5 μL of deionized water three times and let the sample dry for 5 min before TEM imaging. We imaged using a ThermoFisher Tecnai G2 Biotwin TEM (Cat# Tecnai12BT) at 80kV with an AMT side-mount XR80 8-megapixel digital camera. **Figure S2** shows TEM images of aSyn-induced aggregates without MMP1 treatment. Note that we formed the aggregates and fibrils in the presence of other *E. coli* proteins, in contrast to fibrils formed with pure aSyn[1].

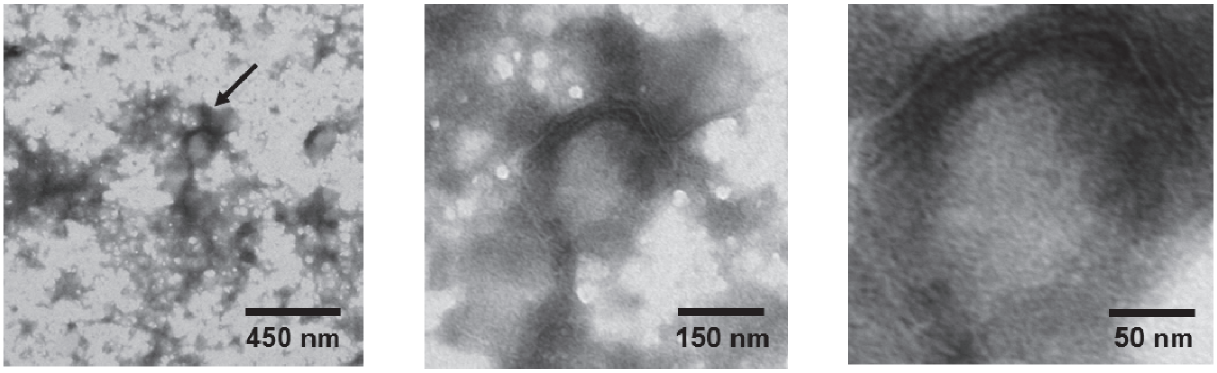

Figure S2. TEM images of aSyn-induced aggregates stained with uranyl acetate. The same field of view imaged at progressively higher resolutions show a mesh of aggregates (left panel), fibrillar structure (middle panel), and individual fibrils in a bundle (right panel). The arrow in the left panel indicates the hook-shaped structure that we imaged at higher resolutions.

Best-fit parameters for experiments. We fitted a sum of two Gaussians to the experimental histograms in **Figure 1**. The best-fit parameters are in **Table S1A**. The fit parameters b1 and b2 are the two states, S1 and S2, respectively. We fitted exponential and power-law distributions to the experimental autocorrelations in **Figure 1.** Power law distribution does not fit the experimental autocorrelations. The best-fit parameters for the exponential fits are in **Table S1B**. The kinetic rates of interconversion between S1 and S2 are in **Table S1C**. The error bars represent the standard errors of the mean.

Table S1. Best-fit parameters for histograms and autocorrelations in Figure 1.

A Gaussian fit parameters for experimental histograms

$$y = a_1 \times e^{-\frac{(x-b_1)^2}{c_1^2}} + a_2 \times e^{-\frac{(x-b_2)^2}{c_2^2}}$$

	MMP1 without ligands		MMP1 with tetracycline	
	Active	Inactive	Active	Inactive
a1	6.03±0.05	1.94±0.15	0.64±0.04	1.95±0.07
b1/S1	0.46±0.01	0.51±0.01	0.43±0.01	0.53±0.01
c1	0.08±0.01	0.10± 0.01	0.06±0.01	0.10±0.01
a2	1.87±0.09	6.24±0.22	5.36±0.03	6.52±0.14
b2/S2	0.52±0.01	0.58±0.01	0.59±0.01	0.61±0.01
c2	0.04±0.01	0.06±0.01	0.10±0.01	0.06±0.01

B Exponential fit parameters for correlations

$$C_\tau = d \times \exp^{-e \times \tau} + f$$

	MMP1 without ligands		MMP1 with tetracycline	
	Active	Inactive	Active	Inactive
d	0.20±0.01	0.36±0.01	0.53±0.01	0.53±0.01
e	0.08±0.01	0.06±0.01	0.05±0.01	0.04±0.01
f	-0.01±0.01	-0.01±0.01	-0.01±0.01	-0.01±0.01

C Kinetic rates calculated from histograms and correlations

	MMP1 without ligands		MMP1 with tetracycline	
	Active	Inactive	Active	Inactive
k1 (s^{-1})	0.0107	0.0396	0.0428	0.0231
k2 (s^{-1})	0.0708	0.0196	0.0030	0.0126

Calculation of correlations. We used the following equation to calculate autocorrelations:

$$C(\tau) = \frac{1}{N-\tau} \sum_{t=1}^{N-\tau} \left\{ I(t) - \frac{1}{N-\tau} \sum_{t'=1}^{N-\tau} I(t') \right\} \times \left\{ I(t+\tau) - \frac{1}{N-\tau} \sum_{t'=1+\tau}^{N} I(t') \right\}$$

where $C(\tau)$ is the correlation at lag number τ, N is the number of points in a time series, and $I(t)$ is the value at t. We illustrate the process using a simple time series (*t*,*x*):

(0,2), (1,3), (2,4), (3,1), (4,5), (5,3)

$C(\tau)$ at lag number $\tau = 0$:

In this time series, *N*=6 and the mean=(2+3+4+1+5+3)/6=18/6=3. After subtracting the mean to analyze only the fluctuations, we obtain a new time series:

(0,2-3), (1,3-3), (2,4-3), (3,1-3), (4,5-3), (5,3-3) or (0,-1), (1,0), (2,1), (3,-2), (4,2), (5,0)

The values in the time series after subtracting the mean are -1, 0, 1, -2, 2, 0. We do not need to shift the time series for $\tau = 0$. We can simply multiply the time series by itself element by element and calculate $C(\tau)$ at the lag number $\tau = 0$ as below:

-1, 0, 1, -2, 2, 0

-1, 0, 1, -2, 2, 0

We multiply element by element, add, and average by $N-\tau=6-0=6$ to get:

1+0+1+4+4+0=10, average=10/6=1.67.

Therefore, $C(\tau)$ at lag number $\tau = 0$ is 1.67.

$C(\tau)$ at lag number $\tau=1$:

We need to shift the time series by the lag number 1 and multiply the time series element by element and calculate $C(\tau)$ at the lag number $\tau=1$ as below:

2, 3, 4, 1, 5, 3
3, 4, 1, 5, 3

We subtract the means from each element to obtain the fluctuations. We disregard the last element in the first row above because it is unpaired. For the first row, the mean is (2+3+4+1+5)/5=15/5=3. For the second row, the mean is (3+4+1+5+3)/5=16/5=3.2. After subtracting the means, we get the following time series:

-1, 0, 1, -2, 2
-0.2, 0.8, -2.2, 1.8, -0.2

We multiply element by element, add, and average by $N-\tau=6-1=5$ to get:

0.2+0-2.2-3.6-0.4=-6, average=-6/5=-1.20.

Therefore, $C(\tau)$ at lag number $\tau=1$ is -1.20.

$C(\tau)$ at lag number $\tau = 2$:

We need to shift the time series by the lag number 2 and multiply the time series element by element and calculate $C(\tau)$ at the lag number $\tau = 2$ as below:

2, 3, 4, 1, 5, 3
4, 1, 5, 3

We subtract the means from each element to obtain the fluctuations. We disregard the last two elements in the first row above because they are unpaired. For the first row, the mean is (2+3+4+1)/4=10/4=2.5. For the second row, the mean is (4+1+5+3)/4=13/4=3.25. After subtracting the means, we get the following time series:

-0.5, 0.5, 1.5, -1.5
-0.75, -2.25, 1.75, -0.25

We multiply element by element, add, and average by $N-\tau=6-2=4$ to get:

0.375-1.125+2.625+0.375=2.25, average=2.25/4=0.5625.

Therefore, $C(\tau)$ at lag number $\tau = 2$ is 0.5625. The autocorrelations at different lag numbers are:

(0,1.67), (1,-1.20), (2,0.5625), …

After normalization by dividing the value at $\tau = 0$, we get:

(0,1), (1,-0.72), (2,0.34), …

We have followed this procedure to calculate normalized autocorrelations. For a two-state Poisson process, the decay of autocorrelations is the sum of two kinetic interconversion rates between the two states.

Table S2. Best-fit parameters for simulated histograms and autocorrelations in Figure 2.

Recovered parameters

Gaussian fit parameters for simulated histograms

$$y = a_1 \times e^{-\frac{(x-b_1)^2}{c_1^2}} + a_2 \times e^{-\frac{(x-b_2)^2}{c_2^2}}$$

	Without noise		With noise	
	Active	Inactive	Active	Inactive
a1			6.06±0.01	1.95±0.01
b1/S1	0.46	0.51	0.46±0.01	0.51±0.01
c1			0.08±0.01	0.10±0.01
a2			1.97±0.01	6.25±0.02
b2/S2	0.52	0.58	0.52±0.01	0.58±0.01
c2			0.04±0.01	0.06±0.01

Input parameters

Active MMP1 (No ligand)
S1 = 0.46, S2 = 0.52
$k1 = 0.01\ s^{-1}$, $k2 = 0.07\ s^{-1}$
$k1 + k2 = 0.08\ s^{-1}$
$\sigma1 = 0.08$, $\sigma2 = 0.04$

Inactive MMP1 (No ligand)
S1 = 0.51, S2 = 0.58
$k1 = 0.04\ s^{-1}$, $k2 = 0.02\ s^{-1}$
$k1 + k2 = 0.06\ s^{-1}$
$\sigma1 = 0.10$, $\sigma2 = 0.06$

Power law and exponential fit parameters for correlations

$$C_\tau = (a \times \tau + 1)^{-b}$$

$$C_\tau = d \times \exp^{-e \times \tau} + f$$

	Without noise		With noise	
	Active	Inactive	Active	Inactive
a	0.01±0.01	0.01±0.01	NA	NA
b	1.8±8.8	1.8±8.6	NA	NA

	Without noise		With noise	
	Active	Inactive	Active	Inactive
d	1.02±0.01	1.03±0.01	0.12±0.01	0.29±0.01
e	0.09±0.01	0.06±0.01	0.08±0.01	0.06±0.01
f	-0.02±0.01	-0.03±0.01	-0.01±0.01	-0.01±0.01

Best-fit parameters for simulations. We considered the MMP1 dynamics as a two-state Poisson process and simulated smFRET trajectories assuming that MMP1 undergoes interconversion between two states, S1 and S2, with experimentally determined kinetic rates and noise levels. We considered active MMP1 and active site mutant of MMP1 without ligands (**Figures 1D** and **1F**). We simulated and analyzed 350 smFRET trajectories, each 1000 s long, with the input parameters in **Table S2**. The recovered parameters are on the right side of **Table S2**.

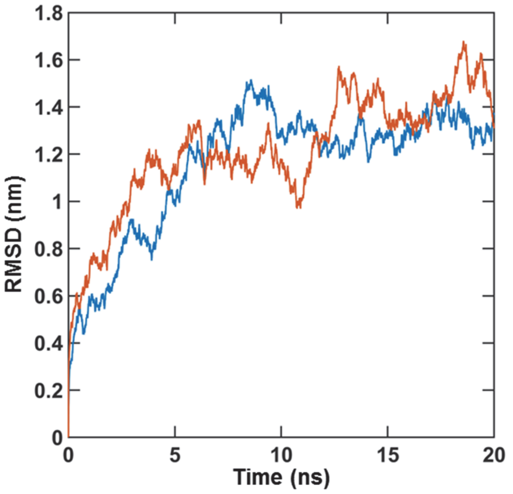

Figure S3. Stabilization of dynamics. RMSD for active (blue) and inactive (orange) MMP1 without ligands at 22 °C.

RMSD stabilization of simulations. We defined the input structure at t=0 as the reference structure and calculated the root-mean-square-displacement (RMSD) to check the simulations' stabilization.

Simulations of MMP1 dynamics stabilize in ~5 ns (**Figure S3**). As such, we simulated 20 ns long dynamics for different conditions.

Calculation of entropy. We calculated the Gray-Level Co-Occurrence Matrix (GLCM)[2] from the two-dimensional correlation plots. Then, we defined the Shannon entropy[3] from the GLCM. We describe the steps for calculating the Shannon entropy of MMP1 conformational dynamics in **Figure S4** using an arbitrary 5×5 matrix, where each element of the matrix has a value between 0 and 3. In other words, we have a 2-bit 5×5 matrix in **Figure S4**. The dimension of GLCM depends on the number of possible values (0, 1, 2, 3), i.e., 4×4 for the 5×5 matrix in **Figure S4**.

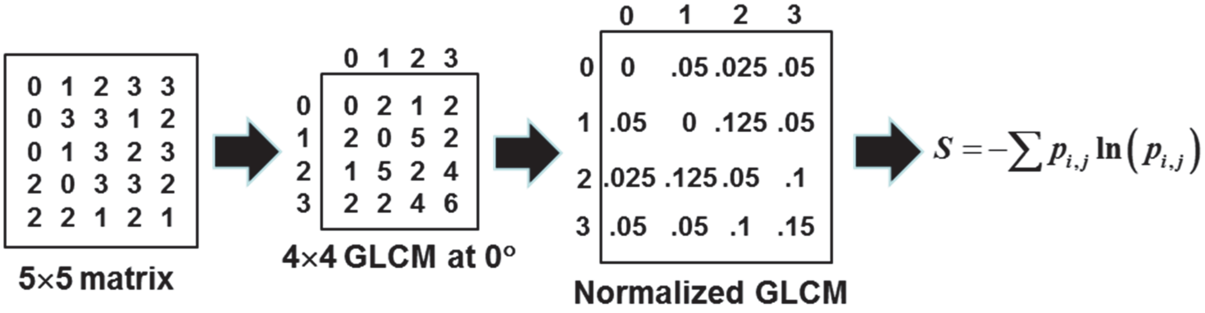

Figure S4. Calculation of GLCM and entropy.

We calculated the GLCM at 0°, i.e., we only considered the next neighbor on the left and right. For example, we calculated the (0,0) element of GLCM by finding the value 0s in the original 5×5 matrix and counting the number of times 0s appear on the left and right. The number is 0, and as such, the (0,0) element of GLCM is 0. To calculate the (0,1) element of GLCM, we found the value 0s in the original 5×5 matrix and counted the number of times 1s appear on the left and right. The number is 2, and as such, the (0,1) element of GLCM is 2. We repeated this process for all the elements of GLCM and obtained the matrix in the middle (**Figure S4**). We calculated the sum of all elements and divided each element by the sum to obtain the normalized GLCM at the right (**Figure S4**). We used $S = -\sum p_{i,j} \ln(p_{i,j})$, where $p_{i,j}$ is the (i,j) element of GLCM to quantify the Shannon entropy. By definition, we considered $\ln(p_{i,j}) = 0$ for $p_{i,j} = 0$. Note that one can calculate GLCM at other angles[2]. For MMP1 conformational dynamics, the dimension of correlation matrices is 367×367. We divided the correlation values between 0 and 1 into 10 bins of width 0.1. As such, the dimension of GLCM for MMP1 dynamics is 10×10.

MD simulations with ions in Figure S5.

Metal Coordination Centre Parameterization. We used a bonded metal model approach to coordinate each metal ion with its corresponding protein coordination center. The six metal ions, two zinc and four calcium ions, with coordinating amino acids were modeled using the AmberTools21 MCPY.py (version 3.0) workflow. We advise the reader to consult the MCPB.py documentation for a complete step-by-step guide[4]. What follows is a brief overview of the steps and the parameters unique to the structure.

The complete coordination geometries of each metal ion coordination shell were first identified with a coordinating distance threshold of 2.8 angstroms using the 4AUO crystal structure of MMP1. Hydrogen atoms were added using the H++ web server[5]. The electronic structure of the

extracted six coordination structures was optimized using GAMESS (US)[6] at a B3LYP/6-31g(d) level in a vacuum to remain consistent with earlier studies[7]. Charge and spin were set appropriately, and the protonation state of histidine was set according to its coordination with an ion. The normal modes and the frequencies were checked for imaginary numbers, and the Hessian was preserved in the output.

Table S3. Best-fit parameters for simulated histograms and correlations in Figure 3.

A Gaussian fit parameters for simulated histograms

$$y = a_1 \times e^{-\frac{(x-b_1)^2}{c_1^2}} + a_2 \times e^{-\frac{(x-b_2)^2}{c_2^2}}$$

Catalytic pocket opening			
	**Pose 1**	**Pose 2**	**Pose 3**
**a1**	2.91±0.10	1.33±0.02	2.20±0.08
**b1/S1 (nm)**	2.54±0.01	2.35±0.01	2.60±0.01
**c1**	0.11±0.01	0.35±0.01	0.17±0.01
**a2**	1.68±0.08	1.57±0.05	1.88±0.22
**b2/S2(nm)**	2.73±0.01	2.52±0.01	2.78±0.01
**c2**	0.12±0.01	0.06±0.01	0.10±0.01

Interdomain distance			
	**Pose 1**	**Pose 2**	**Pose 3**
**a1**	1.82±0.03	1.13±0.03	0.90±0.17
**b1/S1 (nm)**	4.07±0.01	4.38±0.01	4.03±0.01
**c1**	0.21±0.01	0.14±0.01	0.31±0.02
**a2**	0.41±0.03	1.49±0.02	1.73±0.17
**b2/S2(nm)**	5.37±0.02	4.93±0.01	3.99±0.01
**c2**	0.36±0.03	0.26±0.01	0.17±0.01

B Linear correlation fit parameters

$$y_i = b_0 + b_1 \times x_i$$

Linear correlation			
	**Pose 1**	**Pose 2**	**Pose 3**
**b0**	3.25±0.01	1.20±0.03	3.32±0.03
**b1**	-0.14±0.01	0.24±0.01	-0.16±0.01

Force field parameters for Amber ff14SB were generated using the Seminario methods, and restrained electrostatic potential charges were fitted to each structure. The coordinates of a single MMP1 molecule from the 4AUO MMP1 crystal structure were used as a reference, and the residue names for all six metal ions and their respective coordinating side-chains were renamed to match the output files and parameter input file of MCPY.py. The Amber ParmEd program converted the final coordination and topology files suitable for the Gromacs suite.

The parameters and topology data can be found at https://github.com/acnash/MMP1.

Table S4. Best-fit parameters for simulated histograms and autocorrelations in Figure 5.

A Gaussian fit parameters for simulated histograms

$$y = a_1 \times e^{-\frac{(x-b_1)^2}{c_1^2}} + a_2 \times e^{-\frac{(x-b_2)^2}{c_2^2}}$$

MMP1 without ligands		
	Active	Inactive
a1	0.64±0.05	1.79±0.02
b1/S1 (nm)	3.89±0.01	4.25±0.01
c1	0.19±0.02	0.24± 0.01
a2	1.87±0.05	0.58±0.02
b2/S2 (nm)	4.39±0.01	4.74±0.01
c2	0.22±0.01	0.20±0.01

B Exponential fit parameters for correlations

$$C_\tau = d \times \exp^{-e \times \tau} + f$$

MMP1 without ligands		
	Active	Inactive
d	0.26±0.01	0.21±0.01
e	0.006±0.001	0.005±0.001
f	0.67±0.01	0.74±0.01

C Linear correlation fit parameters

$$y_i = b_0 + b_1 \times x_i$$

MMP1 without ligands		
	Active	Inactive
b0	1.19±0.03	2.40±0.03
b1	0.32±0.01	0.04±0.01

Molecular Dynamics Parameters. Atomic step integration was performed using the velocity Verlet algorithm with the time-step of 2 fs using Gromacs 2020.5[8]. The trajectory coordinates and velocities were recorded every 2500 steps (5 ps). Constraints were controlled using the LINCS schema, applied to hydrogen atoms only, with the LINCS iteration set to one with an order of four. The neighbor search was controlled using the Verlet cut-off scheme with the neighbor list updated every 10 steps. Periodic boundary conditions were applied in all directions. Long-range electrostatic interactions were calculated using the fast and smooth Particle-Mesh Ewald (PME) scheme, with a PME interpolation order of 4 and a Fourier spacing of 0.16 nm. A cut-off Van der Waals interactions and short-range Coulomb interactions were set to 1.0 nm, and both were modified with a potential shift. The temperature was set to 295 K for the two groups, water with counter ions and all protein and ligand-related atoms. The time constant for temperature coupling was set to 0.1 ps. Pressure coupling was set to 1 atmosphere and was controlled using the Berendsen followed by Parrinello-Rahman isotropic pressure coupling with a time constant of 2 ps. The compressibility was set to 4.5×10^{-5} bar^{-1}. The reference coordinates were scaled using the center of mass.

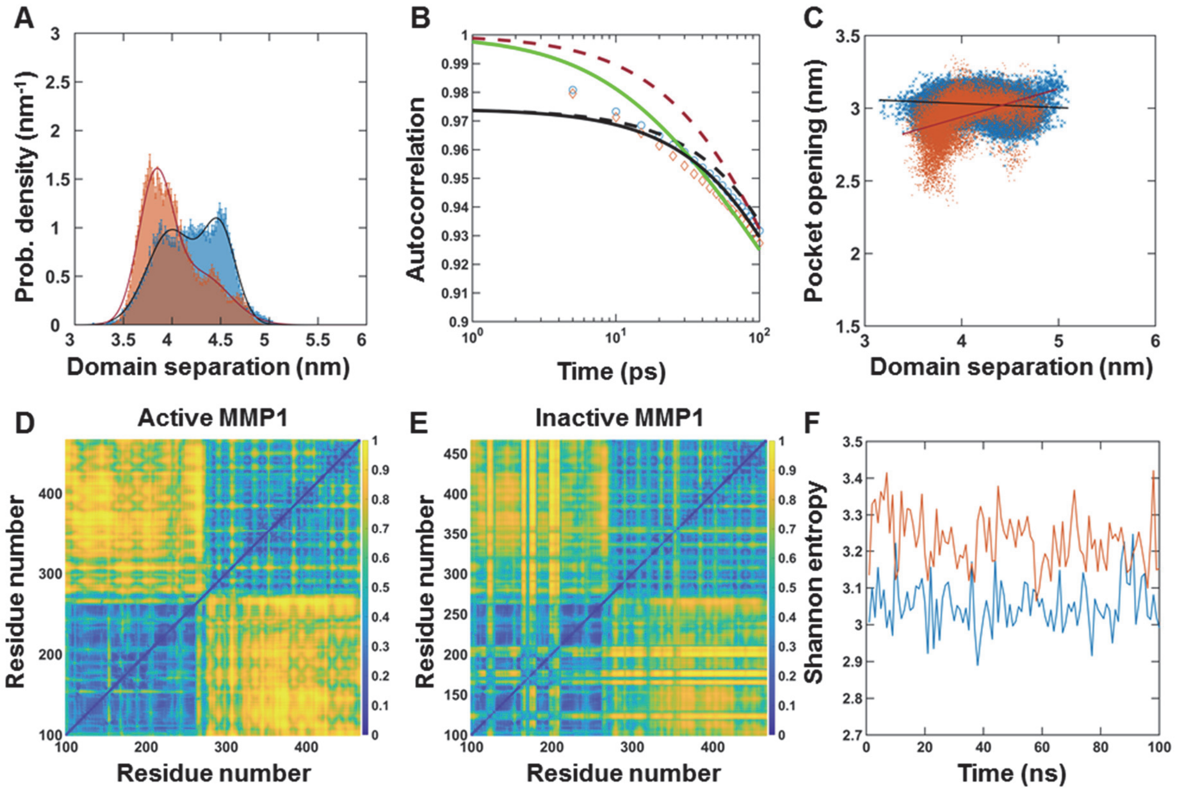

Figure S5. All-atom MD simulation of MMP1 interdomain dynamics of pose 3 with two zinc and four calcium ions at 22 °C. (**A**) Area-normalized histograms of simulated interdomain distance (active: blue; inactive: orange) with best fits to a sum of two Gaussians (solid line). (**B**) Autocorrelations of simulated interdomain distance (active: blue; inactive: orange) with fits to exponentials (active: dashed black line; inactive: solid black line). Power law does not fit autocorrelations (active: dashed red line; inactive: solid green line). (**C**) Linear correlation plots of catalytic pocket opening and interdomain distance. (**D**) and (**E**) Mean correlations between residues for active and inactive MMP1, respectively. Correlations are normalized between 0 (blue) and 1 (yellow). Yellowish colors indicate higher correlations. (**F**) Shannon entropy calculated from correlation plots for active (S= 3.05±0.01, mean±SEM) and inactive (S= 3.23±0.01, mean±SEM). For best-fit parameters, see **Table S5**.

Computational Model Construction. Having performed force field parameterization of the six metal-ion coordination sites using the 4AUO crystal structure as explained, the experimental coordinates of each structure were encoding using the Amber ff14SB force field. Each structure was centered with a distance of at least 1.5 nm from every protein-ligand atom to the boundary of a dodecahedron unit cell, which was then solvated with tip3p water molecules with counter-ions for a neutral charge. Energy minimization using the steepest descent algorithm was performed with a stopping threshold of 800 kJ/mol. Position restraints of 1000 kJ/mol were applied to the alpha-carbon atoms of the MMP-1 and alpha-synuclein ligand.

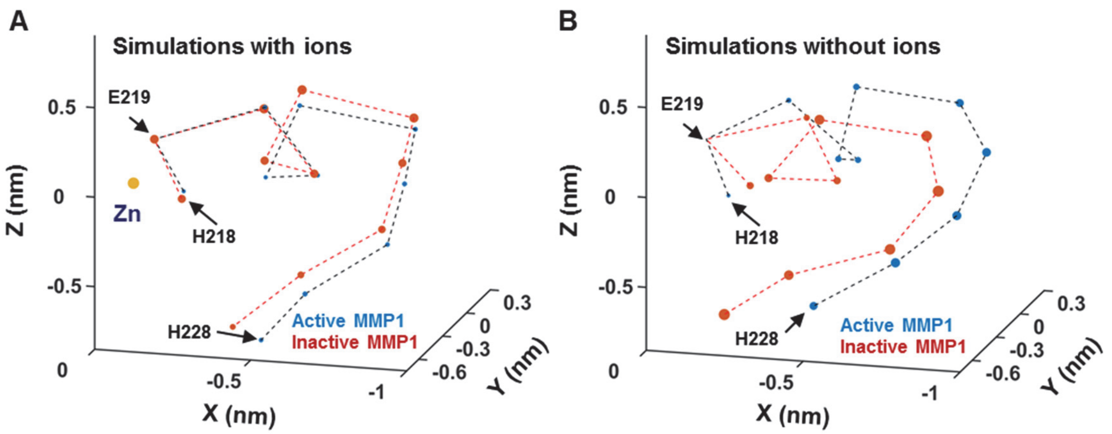

Figure S6. Configuration of the MMP1 catalytic motif at 22 °C with and without metal ions. (**A**) Three-dimensional configurations of the catalytic motif residues for active MMP1 (blue) and inactive MMP1 (orange) at 22 °C with metal ions included during MD simulations. (**B**) Three-dimensional configurations of the catalytic motif residues for active MMP1 (blue) and inactive MMP1 (orange) at 22 °C without considering metal ions during MD simulations.

Table S5. Best-fit parameters for simulated histograms and autocorrelations in Figure S4.

A Gaussian fit parameters for simulated histograms

$$y = a_1 \times e^{-\frac{(x-b_1)^2}{c_1^2}} + a_2 \times e^{-\frac{(x-b_2)^2}{c_2^2}}$$

MMP1 without ligands		
	Active	Inactive
a1	0.97±0.02	1.46±0.11
b1/S1 (nm)	4.01±0.02	3.83±0.01
c1	0.37±0.02	0.26± 0.01
a2	0.91±0.05	0.49±0.04
b2/S2 (nm)	4.50±0.01	4.31±0.07
c2	0.23±0.01	0.42±0.07

B Exponential fit parameters for correlations

$$C_\tau = d \times \exp^{-e \times \tau} + f$$

MMP1 without ligands		
	Active	Inactive
d	0.20±0.01	0.11±0.01
e	0.002±0.001	0.005±0.001
f	0.78±0.01	0.87±0.01

C Linear correlation fit parameters

$$y_i = b_0 + b_1 \times x_i$$

MMP1 without ligands		
	Active	Inactive
b0	3.15±0.01	2.16±0.01
b1	0.03±0.01	0.19±0.01

Each system was equilibrated for 50 ps using the NVT (fixed particles, volume, and temperature) ensemble at 295 K (22 °C) using the velocity rescale scheme. The final frame coordinates and

velocities were used for a further 50 ps while preserving the position restraints and switching to the Berendsen pressure coupling (NPT - fixed particles, pressure, and temperature - ensemble). Each simulation was continued for a further 50 ps with position restraints and a switch to the Parrinello-Rahman pressure coupling. Position restraints were removed, and each system was left to evolve for 100 ns.

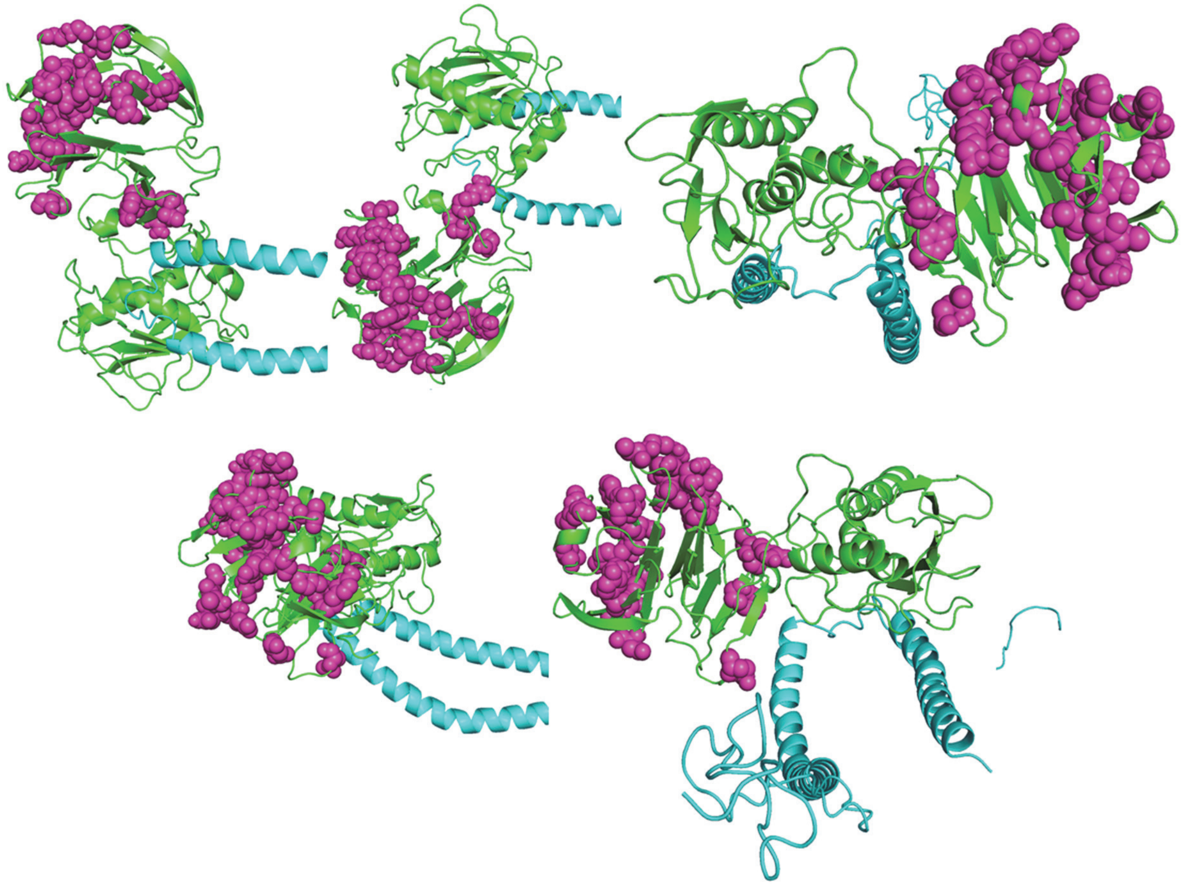

Figure S7. Allosteric residues in MMP1 specific to aSyn. We identified aSyn-specific allosteric residues in MMP1 shown in different orientations of pose 3.

Small molecule virtual screening. An example of Autodock parameters shared amongst ligands:

```
autodock_parameter_version 4.2      # used by autodock to validate parameter set
outlev 1                            # diagnostic output level
intelec                             # calculate internal electrostatics
seed pid time                       # seeds for random generator
ligand_types A C HD N OA SA         # atoms types in ligand
fld MMP1aSyn01.maps.fld             # grid_data_file
map MMP1aSyn01.A.map                # atom-specific affinity map
map MMP1aSyn01.C.map                # atom-specific affinity map
map MMP1aSyn01.HD.map               # atom-specific affinity map
map MMP1aSyn01.N.map                # atom-specific affinity map
map MMP1aSyn01.OA.map               # atom-specific affinity map
map MMP1aSyn01.SA.map               # atom-specific affinity map
elecmap MMP1aSyn01.e.map            # electrostatics map
```

```
desolvmap MMP1aSyn01.d.map                # desolvation map
move ZINC000001536779.pdbqt               # small molecule – **change for each ligands**
about -3.608 -2.104 1.030                 # small molecule center
tran0 random                              # initial coordinates/A or random
quaternion0 random                        # initial orientation
dihe0 random                              # initial dihedrals (relative) or random
torsdof 6                                 # torsional degrees of freedom
rmstol 2.0                                # cluster_tolerance/A
extnrg 1000.0                             # external grid energy
e0max 0.0 10000                           # max initial energy; max number of retries
ga_pop_size 150                           # number of individuals in population
ga_num_evals 2500000                      # maximum number of energy evaluations
ga_num_generations 27000                  # maximum number of generations
ga_elitism 1                              # top individuals to survive to next generation
ga_mutation_rate 0.02                     # rate of gene mutation
ga_crossover_rate 0.8                     # rate of crossover
ga_window_size 10                         #
ga_cauchy_alpha 0.0                       # Alpha parameter of Cauchy distribution
ga_cauchy_beta 1.0                        # Beta parameter Cauchy distribution
set_ga                                    # set the above parameters for GA or LGA
sw_max_its 300                            # iterations of Solis & Wets local search
sw_max_succ 4                             # consecutive successes before changing rho
sw_max_fail 4                             # consecutive failures before changing rho
sw_rho 1.0                                # size of local search space to sample
sw_lb_rho 0.01                            # lower bound on rho
ls_search_freq 0.06                       # probability of performing local search on individual
set_psw1                                  # set the above pseudo-Solis & Wets parameters
unbound_model bound                       # state of unbound ligand
ga_run 10                                 # do this many hybrid GA-LS runs
analysis                                  # perform a ranked cluster analysis
```

Table S6. Best-fit parameters for correlation histograms in Figure 6.

Gaussian fit parameters for correlation histograms

$$y = a_1 \times e^{-\frac{(x-b_1)^2}{c_1^2}} + a_2 \times e^{-\frac{(x-b_2)^2}{c_2^2}}$$

	Free	Pose 3
**a1**	1.60±0.03	1.23±0.04
**b1**	0.52±0.01	0.44±0.01
**c1**	0.21±0.01	0.19± 0.01
**a2**	1.62±0.08	1.87±0.05
**b2**	0.82±0.01	0.75±0.01
**c2**	0.14±0.01	0.18±0.01

References

1 de Oliveira, G. A. & Silva, J. L. Alpha-synuclein stepwise aggregation reveals features of an early onset mutation in Parkinson's disease. *Communications Biology* **2**, 1-13 (2019).

2 Haralick, R. M., Shanmugam, K. & Dinstein, I. H. Textural features for image classification. *IEEE Transactions on Systems, Man, and Cybernetics*, 610-621 (1973).

3 Shannon, C. E. A mathematical theory of communication. *The Bell System Technical Journal* **27**, 379-423 (1948).

4 Li, P. & Merz Jr, K. M. (ACS Publications, 2016).

5 Anandakrishnan, R., Aguilar, B. & Onufriev, A. V. H++ 3.0: automating p K prediction and the preparation of biomolecular structures for atomistic molecular modeling and simulations. *Nucleic Acids Research* **40**, W537-W541 (2012).

6 Barca, G. M. *et al.* Recent developments in the general atomic and molecular electronic structure system. *The Journal of Chemical Physics* **152**, 154102 (2020).

7 Nash, A., Birch, H. L. & de Leeuw, N. H. Mapping intermolecular interactions and active site conformations: from human MMP-1 crystal structure to molecular dynamics free energy calculations. *Journal of Biomolecular Structure and Dynamics* **35**, 564-573 (2017).

8 Abraham, M. J. *et al.* GROMACS: High performance molecular simulations through multi-level parallelism from laptops to supercomputers. *SoftwareX* **1**, 19-25 (2015).
